# Mercury levels in hair are associated with reduced neurobehavioral performance and altered brain structures in young adults

**DOI:** 10.1038/s42003-022-03464-z

**Published:** 2022-06-02

**Authors:** Hikaru Takeuchi, Yuka Shiota, Ken Yaoi, Yasuyuki Taki, Rui Nouchi, Ryoichi Yokoyama, Yuka Kotozaki, Seishu Nakagawa, Atsushi Sekiguchi, Kunio Iizuka, Sugiko Hanawa, Tsuyoshi Araki, Carlos Makoto Miyauchi, Kohei Sakaki, Takayuki Nozawa, Shigeyuki Ikeda, Susumu Yokota, Daniele Magistro, Yuko Sassa, Ryuta Kawashima

**Affiliations:** 1 Division of Developmental Cognitive Neuroscience, Institute of Development, Aging and Cancer, Tohoku University, Sendai, Japan; 2 United Graduate School of Child Development, Osaka University, Kanazawa University, Hamamatsu University School of Medicine, Chiba University and University of Fukui, Kanazawa, Japan; 3 Research Center for Child Mental Development, Kanazawa University, Kanazawa, Japan; 4 Division of Medical Neuroimaging Analysis, Department of Community Medical Supports, Tohoku Medical Megabank Organization, Tohoku University, Sendai, Japan; 5 Department of Radiology and Nuclear Medicine, Institute of Development, Aging and Cancer, Tohoku University, Sendai, Japan; 6 Creative Interdisciplinary Research Division, Frontier Research Institute for Interdisciplinary Science, Tohoku University, Sendai, Japan; 7 Human and Social Response Research Division, International Research Institute of Disaster Science, Tohoku University, Sendai, Japan; 8 School of Medicine, Kobe University, Kobe, Japan; 9 Division of Clinical research, Medical-Industry Translational Research Center, Fukushima Medical University School of Medicine, Fukushima, Japan; 10 Department of Human Human Brain Brain Science, Institute of Development, Aging and Cancer, Tohoku University, Sendai, Japan; 11 Division of Psychiatry, Tohoku Medical and Pharmaceutical University, Sendai, Japan; 12 Department of Behavioral Medicine, National Institute of Mental Health, National Center of Neurology and Psychiatry, Tokyo, Japan; 13 Department of Psychiatry, Tohoku University Graduate School of Medicine, Sendai, Japan; 14 ADVANTAGE Risk Management Co., Ltd, Tokyo, Japan; 15 Department of Advanced Brain Science, Institute of Development, Aging and Cancer, Tohoku University, Sendai, Japan; 16 Research Institute for the Earth Inclusive Sensing, Tokyo Institute of Technology, Tokyo, Japan; 17 Department of Ubiquitous Sensing, Institute of Development, Aging and Cancer, Tohoku University, Sendai, Japan; 18 Division for Experimental Natural Science, Faculty of Arts and Science, Kyushu University, Fukuoka, Japan; 19 National Centre for Sport and Exercise Medicine (NCSEM), The NIHR Leicester-Loughborough Diet, Lifestyle and Physical Activity Biomedical Research Unit, School of Sport, Exercise, and Health Sciences, Loughborough University, Loughborough, England

**Keywords:** Intelligence, Risk factors

## Abstract

The detrimental effects of high-level mercury exposure on the central nervous system as well as effects of low-level exposure during early development have been established. However, no previous studies have investigated the effects of mercury level on brain morphometry using advance imaging techniques in young adults. Here, utilizing hair analysis which has been advocated as a method for biological monitoring, data of regional gray matter volume (rGMV), regional white matter volume (rWMV), fractional anisotropy (FA) and mean diffusivity (MD), cognitive functions, and depression among 920 healthy young adults in Japan, we showed that greater hair mercury levels were weakly but significantly associated with diminished cognitive performance, particularly on tasks requiring rapid processing (speed measures), lower depressive tendency, lower rGMV in areas of the thalamus and hippocampus, lower rWMV in widespread areas, greater FA in bilaterally distributed white matter areas overlapping with areas of significant rWMV reductions and lower MD of the widely distributed gray and white matter areas particularly in the bilateral frontal lobe and the right basal ganglia. These results suggest that even normal mercury exposure levels in Japan are weakly associated with differences of brain structures and lower neurobehavioral performance and altered mood among young adults.

## Abstract (web summary)

An analysis of cognitive and brain structural data from over 900 Japanese participants reveals a relationship between increased hair mercury levels and reduced cognitive performance and reductions in gray and white matter volume.

## Introduction

Mercury is a highly neurotoxic heavy metal, and the deleterious effects of high-level exposure to alkyl compounds including methylmercury (produced by microorganisms from environmental mercury) has been well described due to recent public health disasters in Minamata, Japan, and in Iraq^1^. Methylmercury bioaccumulation in fish and subsequent human consumption is the main mechanism of exposure among the general population^2^. The central nervous system is particularly vulnerable to the deleterious effects of methylmercury^3^, which include a variety of neurocognitive deficits, especially of the sensory systems, changes in mental health status including depression, and even cerebral palsy in children exposed to methylmercury in utero^4^.

While the effects of high-level mercury exposure are easily detected, the effects of daily low-level exposure on neurocognitive functions and affective states are still controversial. A previous study of 384 middle-aged adults found that high blood mercury level was associated with lower cognitive functions, particularly those requiring rapid processing^5^. Further, multiple large-scale epidemiological studies have reported that low-level maternal mercury exposure is associated with poor cognitive performance among offspring later in life^6^. However, another previous study of 474 participants concluded that the data could not provide strong evidence for an association between higher blood mercury levels and poor neurobehavioral performance in older adults^7^. Surprisingly, a cross-sectional study of 6911 adults found that higher total blood mercury was associated with lower odds of depression in older subgroups^8^. Alternatively, several other epidemiological studies failed to detect an association between dental amalgam or urinary mercury with depression^9,10^. Numerous basic non-human neuroscientific studies have confirmed the neurotoxicity of mercury, including particularly devastating effects on myelin and axons^11^, though other than these, the mechanisms of neurotoxicity of mercury is thought to be diverse^12^.

However, three major issues remain unresolved. (1) While previous studies have investigated the associations of low-level body mercury with cognitive functions and affective states in adults, no previous studies have investigated the associations of low-level body mercury level with brain morphometry using advanced neuroimaging techniques. (2) While the deleterious effects of low-level mercury on the developing brain are well established, particularly of in utero exposure, effects on adults have been inconsistent across studies. One possible reason is that in adults, the blood-brain barrier is fully developed and can prevent or slow methylmercury accumulation in the central nervous system^13^. (3) Previous large sample studies have used blood or urinary mercury levels for investigating the effects of body mercury level, but these measures are relatively sensitive to short-term variations in exposure. In contrast, hair levels of mercury are not subject to rapid fluctuations due to variations in intake. For these reasons, hair is claimed to be by far the preferred and accepted matrix for biomonitoring of mercury (for review, see^14^) and a reliable representative tissue for biomonitoring of mercury^15^.

The purpose of this study is to resolve these issues. We aimed to reveal the associations of hair mercury levels with cognitive functions, depression, and brain structural measures, particularly white matter structures, in young adults.

Based on the aforementioned findings, we hypothesized that hair mercury level is associated with impaired cognitive functions (particularly processing speed), greater levels of depression, lower regional gray matter volume (rGMV), lower regional white matter volume (rWMV), and lower fractional anisotropy (FA), a diffusion tensor imaging (DTI) metric reflecting the microstructural property of white matter, including myelination^16^), and with higher mean diffusivity (MD), another DTI metric reflecting the effects of various tissue components on the free diffusion of water molecules, in both gray and white matter. Gray and white matter volumetric measures as well as FA and MD measures have been widely used to investigate the effects of the various toxin on neural structure^17–19^. Regional WMV and FA are believed to reflect distinct white matter properties, especially in deep white matter areas^20^, and to associate with different cognitive measures^21^. Various tissue properties, such as the densities and shapes of synapses, capillaries, macromolecular proteins, neurons, glia, myelin, and axons^22,23^, reduced free water diffusion and thus lower MD. In addition to revealing important properties of white matter, MD can reveal unique properties of gray matter. For instance, MD in the dopaminergic system may be characteristically associated with altered dopaminergic system properties (see our recent review^19^). Here we examined the effects of mercury on gray and white matter structure by combining multiple neuroimaging metrics.

The neurotoxicity of mercury is a matter of public health concern. The risk of eating fish contaminated by mercury was raised by the US Food and Drug Administration^24^. This risk is important given the rising interest in the health benefits of fish and fish products, and is particularly critical in countries such as Japan where fish consumption rates and average body mercury levels are already relatively high^25^. Thus, the investigations of the effects of the neurocognitive mechanisms in the present sample is important.

## Results

### Basic data

The data of 920 healthy right-handed individuals (561 males and 359 females, mean age, 20.7 years, age range: 18–27) were used to examine associations among hair mercury levels, measures of brain structure, and neuropsychological test results.

The mean and standard deviation of age, general intelligence test score, Beck Depression Inventory score, and hair Hg levels (raw and log) are presented in Supplementary Table 1. The average hair mercury level was similar to that measured in a previous study in the modern era of Japan and relatively high compared to countries with lower fish consumption^26^. The distribution of raw Hg levels in male and female subjects is presented in Supplementary Fig. 1. Average Beck Depression Inventory score in this study cohort was within the normal range (8.14, standard deviation: 6.39), although a few high scores were found (range: 0–31). Note, however, that the Beck Depression Inventory -II score distribution for the normal population excluding subjects receiving psychiatric treatments and/or with substance dependence still includes high scores overlapping with those of psychiatric patients^27^.

#### Associations between hair Hg levels and psychological variables

**Fig. 1:**
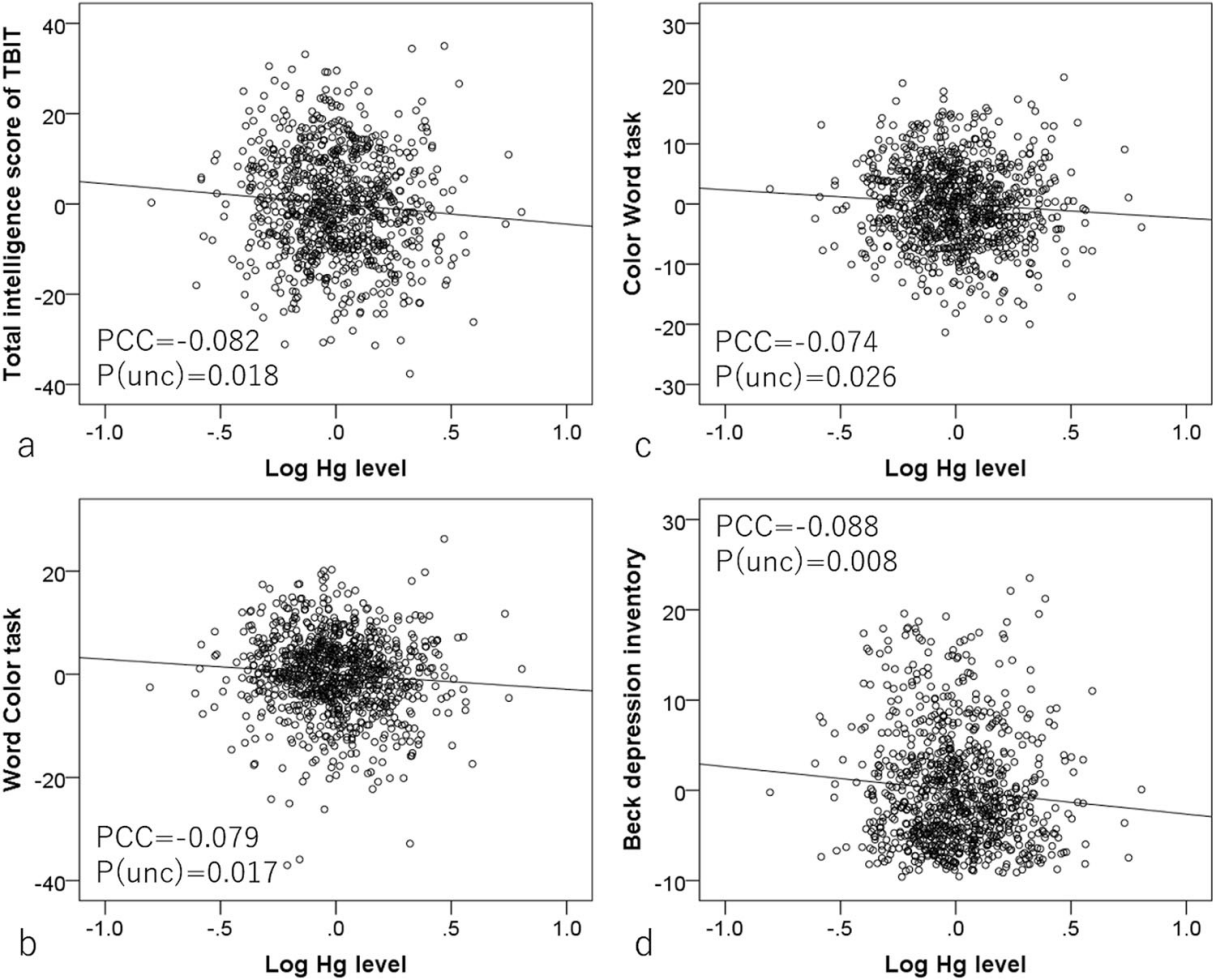
Associations between hair mercury levels and psychological variables. Partial residual plots with the trend lines depicting the associations between residuals of psychological variables and residuals of the logarithms of hair mercury levels with other confounding factors controlled. PCC indicates partial correlation coefficients. Greater hair mercury levels (log values) were significantly associated with (**a**) lower total intelligence score on the TBIT, (**b**) lower performance on the Word-Color task, (**c**) lower performance on the Color-Word task, and (**d**) lower score on the Beck depression inventory.

Partial correlation analyses controlling for confounding variables [sex, age, self-reported height, self-reported weight, and body mass index (BMI, calculated from the self-reported height and self-reported weight), annual family income, parents’ highest educational qualifications (measured as reported previously^28^), fatty fish intake, and intake of fish with less fat] and multiple comparisons revealed that the logarithm of hair mercury level was significantly and negatively correlated with total intelligence score on the Tanaka B-type intelligence test (TBIT), perception factor score on the TBIT, Word-Color task score, Color-Word task score, and Beck Depression Inventory score but not with the other psychological variables measured (Fig. 1). The effect sizes of significant associations were generally weak and the absolute values of partial correlation coefficients were all smaller than 0.09. The results of all psychological analyses are presented in Table 1.

**Table 1:** Results of partial correlation analyses performed using psychological variables and hair mercury level after correcting for confounding variables.

Dependent variables			Hg level	
	N	Partial correlation coefficient	*p (uncorrected)*	*p (FDR*^a^*)*
Raven’s Advance Progressive Matrices	920	−0.039(−0.103~0.025)	0.236	0.118
Total intelligence score of TBIT^b^	843	−0.080(−0.145~−0.014)	0.018	0.034*
Perception score of TBIT	843	−0.075(−0.143~−0.008)	0.028	0.034*
Spatial relation factor of TBIT	849	−0.051(−0.116~0.014)	0.122	0.087
Reasoning factor of TBIT	849	−0.063(−0.127~0.001)	0.053	0.053
Simple arithmetic	661	−0.052(−0.127~0.023)	0.173	0.104
Complex arithmetic	661	0.003(−0.073~0.079)	0.942	0.377
Word-Color task	920	−0.079(−0.143~−0.014)	0.017	0.034*
Color-Word task	920	−0.074(−0.138~−0.009)	0.026	0.034*
Reverse Stroop task	918	−0.014(−0.079~0.050)	0.665	0.285
Stroop task	918	−0.061(−0.125~0.004)	0.065	0.056
Reading comprehension	837	−0.052(−0.120~0.016)	0.131	0.087
S-A creativity test	920	0.041(−0.023~0.105)	0.211	0.115
Digit span	915	−0.037(−0.101~0.027)	0.260	0.120
Beck depression Inventory	917	−0.087(−0.152~−0.023)	0.008	0.034*

^a^False discovery rate. ^b^Tanaka B-type intelligence test.

*p < 0.05, corrected for FDR.

#### Associations of hair Hg levels with rGMV and rWMV

**Fig. 2:**
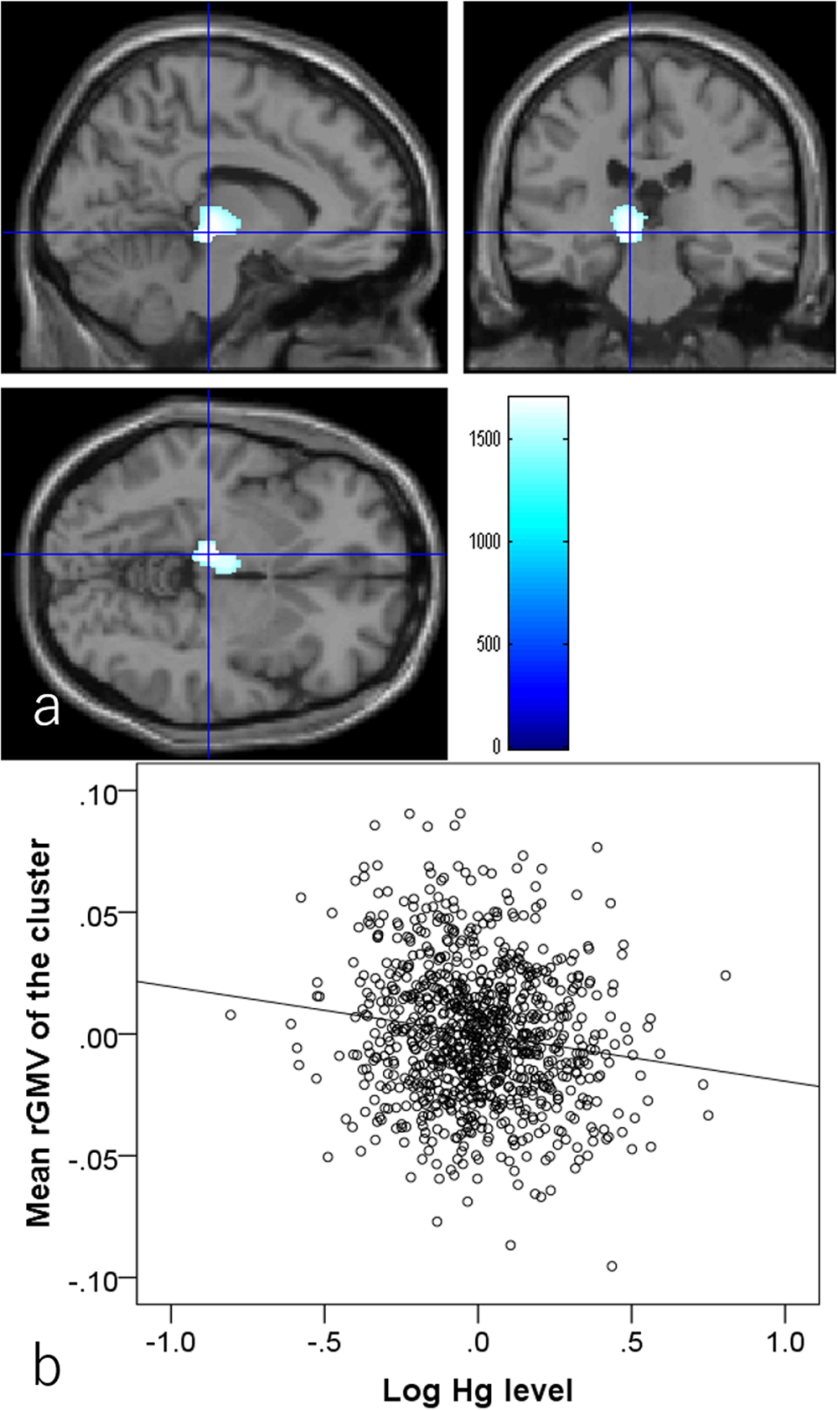
Associations of hair mercury level with regional gray matter volume (rGMV)(*N* = 920). Greater hair mercury level was significantly associated with lower rGMV in the area of the left thalamus and left hippocampus. Colored bars indicate threshold-free cluster enhancement (TFCE) scores. TFCE score is unit-free and reflects both voxel’s height and the sum of the spatially contiguous voxels supporting it; therefore, it reflects both the strength and extent of effects. All results are overlaid on a “single-subject T1” SPM8 image. **a** Regions with significant results. The results shown were obtained using a threshold *P* < 0.05, corrected for multiple comparisons based on 5000 permutations using the TFCE score. **b** A partial residual plot with the trend line depicting the associations between residuals of mean rGMV in the significant cluster and the residuals of the logarithms of hair mercury levels with other confounding factors controlled.

**Fig. 3:**
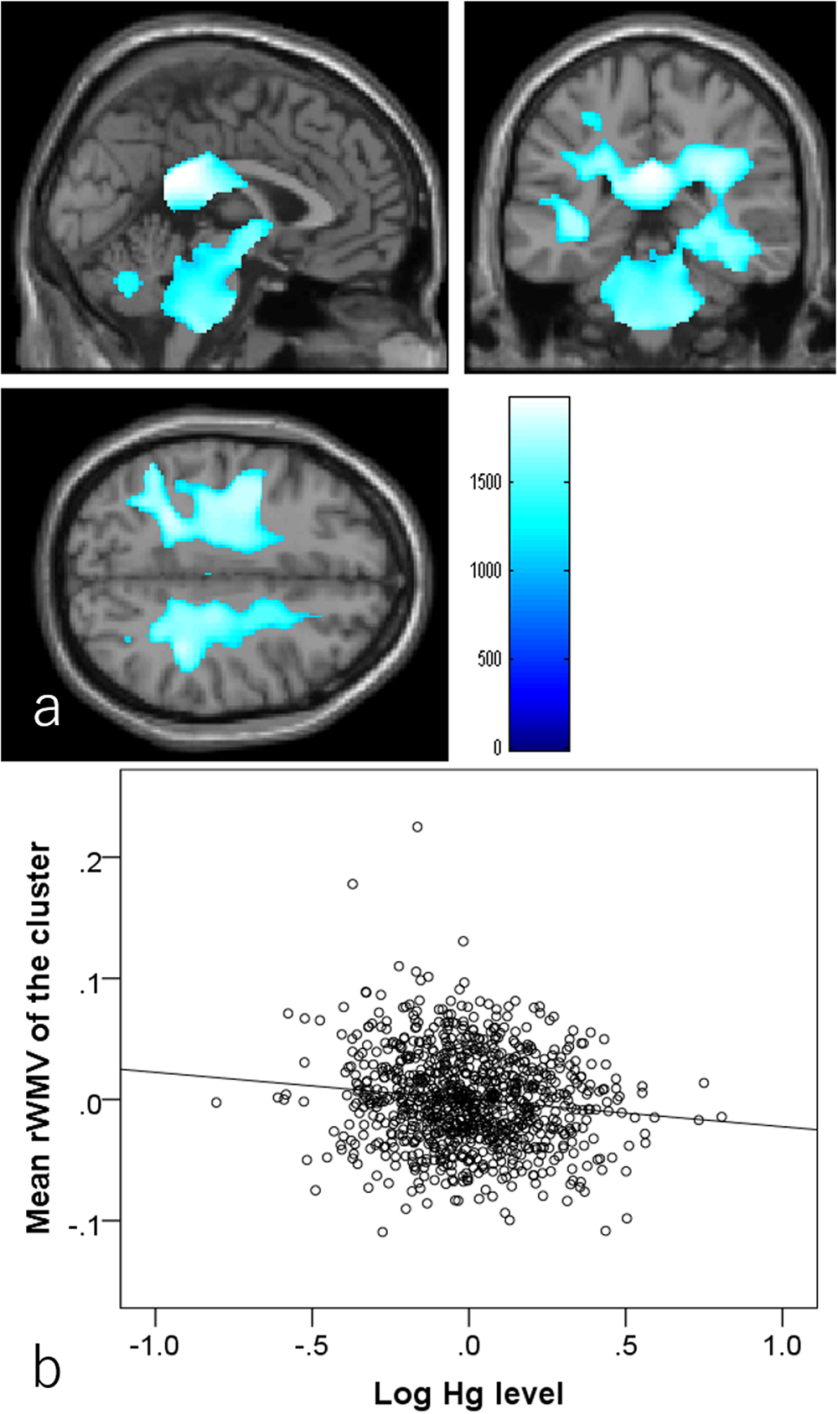
Associations of hair mercury level with regional white matter volume (rWMV)(*N* = 920). Greater hair mercury level was significantly associated with lower rWMV in widespread white matter areas. Colored bars indicate TFCE scores. TFCE score is unit-free and reflects both voxel’s height and the sum of the spatially contiguous voxels supporting it; therefore, it reflects both the strength and extent of effects. All results are overlaid on a “single-subject T1” SPM8 image. **a** Regions with significant results. The results shown were obtained using a threshold *P* < 0.05, corrected for multiple comparisons based on 5000 permutations using the TFCE score. **b** A partial residual plot with the trend line depicting the associations between residuals of mean rWMV in the largest significant cluster and the residuals of the logarithms of hair mercury levels with other confounding factors controlled.

Whole-brain multiple regression analysis controlling for the same variables as in psychological analyses revealed that greater hair Hg level was associated with lower rGMV in a significant cluster mainly located in the left thalamus and extending into the left hippocampus (Fig. 2; Montreal Neurological Institute coordinates: x, y, z = −9, −25.5, −4.5, T score = 4.63, *p* = 0.031, corrected for multiple comparison [family wise error (F.W.E), threshold-free cluster enhancement (TFCE), permutation), TFCE score = 1699.25]. Whole-brain multiple regression analysis also showed that greater hair Hg level was associated with lower rWMV in widespread bilateral white matter areas including the cerebellar peduncle, pontine crossing tract, corticospinal tract, body and splenium of the corpus callosum, lemniscus, fornix and cingulum, internal and external capsule, anterior, posterior, and superior corona radiata, posterior thalamic radiation, sagittal stratum, stria terminalis, superior longitudinal fasciculus, superior fronto-occipital fasciculus, inferior fronto-occipital fasciculus, uncinate fasciculus, and tapetum (Fig. 3 and Table 2). Again, the effect sizes of these significant associations between hair mercury levels and mean values of significant clusters were generally weak, and the absolute values of partial correlation coefficients all <0.140.

**Table 2:** Brain regions with significant associations between greater hair mercury levels and lower rWMV.

	Included large bundles* (number of significant voxels in left and right side of each anatomical area)	x	y	z	T score	TFCE value	Corrected *p* value (FWE)	Cluster size (voxels)
1	Middle cerebellar peduncle (4114)/Pontine crossing tract (522)/Body of corpus callosum (1427)/Splenium of corpus callosum (2697)/Fornix (76)/Corticospinal tract (L:340, R:473)/Medial lemniscus (L:265, R:264)/Inferior cerebellar peduncle (L:265, R:172)/Superior cerebellar peduncle (L:270, R:180)/Cerebral peduncle (L:694, R:817)/Anterior limb of internal capsule (L:152, R:496)/Posterior limb of internal capsule (L:8, R:175)/Retrolenticular part of internal capsule (L:238, R:321)/Anterior corona radiata (L:103, R:107)/Superior corona radiata (L:1541, R:1757)/Posterior corona radiata (L:1004, R:953)/Posterior thalamic radiation (L:420, R:479)/Sagittal stratum (L:235, R:531)/External capsule (L:49, R:59)/Cingulum (L:188, R:168)/Heschl gyrus (L:43, R:168)/Stria terminalis (L:30, R:176)/Superior longitudinal fasciculus (L:1568, R:1294)/Superior fronto-occipital fasciculus (L:107, R:102)/Inferior fronto-occipital fasciculus (L:5, R:18)/Uncinate fasciculus (L:58, R:85)/Tapatum (L:168, R:25)/	0	−42	22.5	4.03	1971.92	0.009	56116
2	Posterior thalamic radiation (L:3)/	−36	−73.5	1.5	3.41	1105.06	0.044	143

*The anatomical labels and significant clusters of major white matter fibers were determined using the ICBM DTI-81 Atlas (http://www.loni.ucla.edu/).

#### Associations between hair Hg levels and FA

**Fig. 4:**
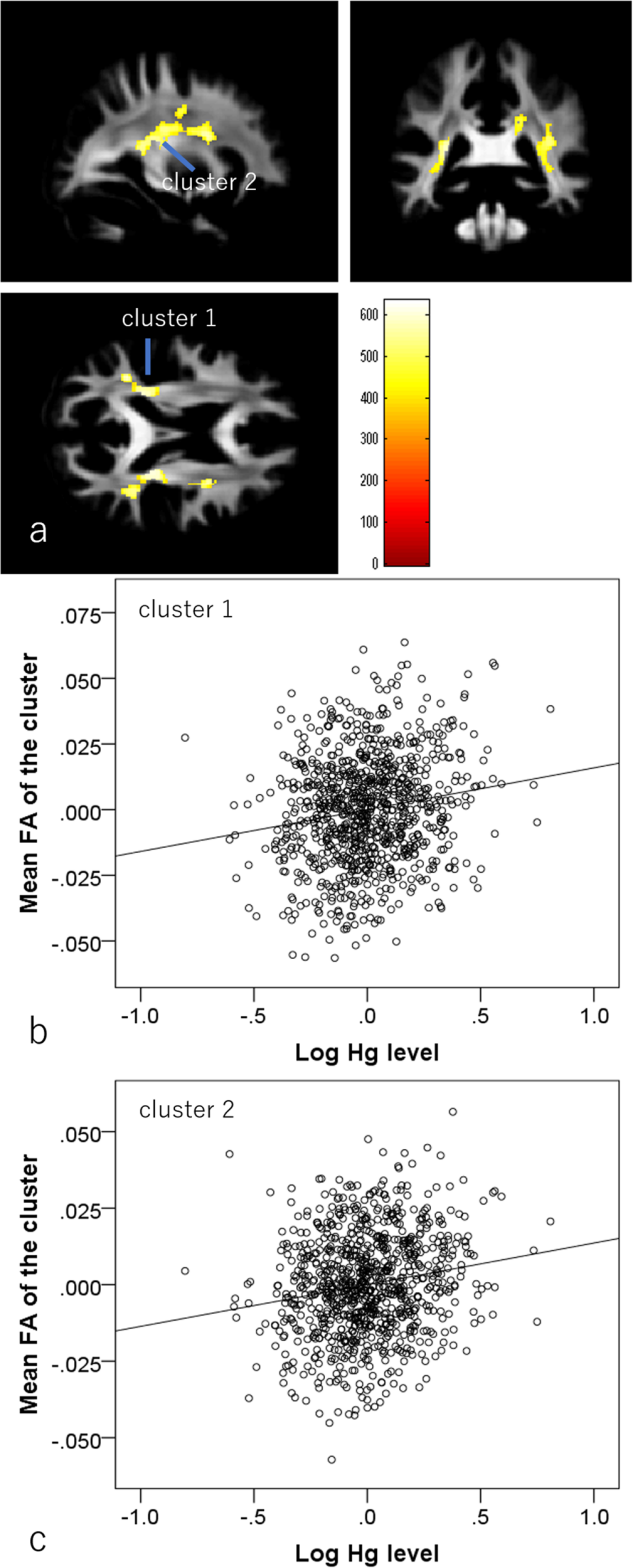
Associations of hair mercury level with fractional anisotropy (FA)(*N* = 919). Greater hair mercury level was significantly associated with greater FA in widespread bilateral white matter areas. Colored bars indicate TFCE scores. TFCE score is unit-free and reflects both voxel’s height and the sum of the spatially contiguous voxels supporting it; therefore, it reflects both the strength and extent of effects. All results are overlaid on the mean preprocessed (including normalization) but unsmoothed FA images of participants from whom the DARTEL template was created (meaning that this mean image is in the normalized space). **a** Regions with significant results. The results shown were obtained using a threshold *P* < 0.05, corrected for multiple comparisons based on 5000 permutations using the TFCE score. **b**, **c** Partial residual plots with the trend line depicting the associations between residuals of the logarithms of hair mercury levels and (**b**) residuals of mean FA in the significant cluster of the left posterior areas and (**c**) residuals of mean FA in the significant cluster of right middle white matter areas with other confounding factors controlled.

Whole-brain multiple regression analysis controlling for the same variables as in psychological analyses showed that higher hair Hg level was associated with greater FA in an anatomical cluster spread across the left internal capsule, left posterior corona radiata, left posterior thalamic radiation, left superior longitudinal fasciculus, and left tapetum, another anatomical cluster spread across the right internal capsule, right superior corona radiata, right posterior corona radiata, right posterior thalamic radiation, right external capsule, and right superior longitudinal fasciculus, and a third anatomical cluster spread across the splenium of the corpus callosum and the right posterior corona radiata (Fig. 4 and Table 3). Similar to other neuroimaging metrics, the effect sizes of significant associations between hair mercury levels and mean values of significant clusters were generally weak, and absolute values of partial correlation coefficients were all smaller than 0.180.

**Table 3:** Brain regions with significant associations between greater hair mercury levels and greater fractional anisotropy.

	Included large bundles* (number of significant voxels in left and right side of each anatomical area)	x	y	z	T score	TFCE value	Corrected *p* value (FWE)	Cluster size (voxel)
1	Posterior limb of internal capsule (L:18)/Retrolenticular part of internal capsule (L:97)/Posterior corona radiata (L:161)/Posterior thalamic radiation (L:308)/Superior longitudinal fasciculus (L:119)/Tapatum (L:58)/	−36	−45	7.5	4.48	634.12	0.004	889
2	Posterior limb of internal capsule (R:30)/Retrolenticular part of internal capsule (R:118)/Superior corona radiata (R:163)/Posterior corona radiata (R:121)/Posterior thalamic radiation (R:72)/External capsule (R:10)/Superior longitudinal fasciculus (R:685)/	33	−12	27	4.14	617.13	0.005	1743
3	Splenium of corpus callosum (105)/Posterior corona radiata (R:111)/	19.5	−42	30	3.85	478.68	0.022	250

*The anatomical labels and significant clusters of major white matter fibers were determined using the ICBM DTI-81 Atlas (http://www.loni.ucla.edu/).

#### Associations between hair Hg levels and MD

**Fig. 5:**
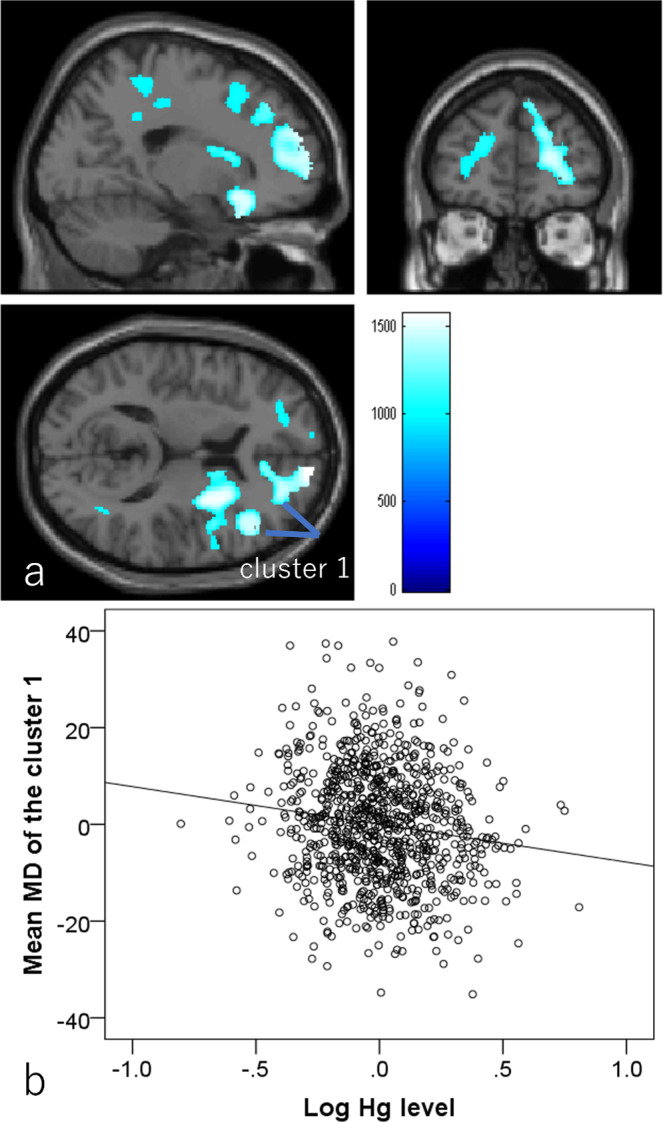
Associations of hair mercury level with mean diffusivity (MD) (*N* = 919). Greater hair mercury level was significantly associated with lower MD in widespread bilateral gray and white matter areas. Colored bars indicate TFCE scores. TFCE score is unit-free and reflects both voxel’s height and the sum of the spatially contiguous voxels supporting it; therefore, it reflects both the strength and extent of effects. All results are overlaid on a single-subject T1-weighted SPM8 image. **a** Regions with significant correlations. The results shown were obtained at a threshold *P* < 0.05, corrected for multiple comparisons based on 5000 permutations using the TFCE score. **b** Partial residual plots with trend line depicting the associations between residuals of log (hair mercury) and residuals of mean MD in the significant cluster spread across the right hemisphere, with other confounding factors controlled.

Whole-brain multiple regression analyses controlling for the same variables as in psychological analyses revealed that higher hair Hg concentration was associated with lower MD in an anatomical cluster that spread over a widespread area of the right lateral and medial frontal cortex, within the supplementary motor cortex, anterior cingulate cortex, and basal ganglia, voxels spread across the right temporal pole, right amygdala, and right anterior temporal gyrus, a cluster spread across the anterior left frontal cortex, and a cluster spread across the right posterior/middle cingulum, right superior parietal lobe, and right angular gyrus (Fig. 5). These extensive clusters of negative correlation did not mostly overlapped with those of negative correlates of rWMV and those of positive correlates of FA, but three were partly overlapped in the white matter areas between the basal ganglia and frontal lobe. Statistical results are summarized in Table 4.

**Table 4:** Brain regions with significant associations between greater hair mercury levels and lower mean diffusivity.

	Included gray matter areas* (number of significant voxels in left and right hemisphere for each anatomical area)	Included large bundles** (number of significant voxels in left and right hemisphere for each anatomical area)	x	y	z	T score	TFCE value	Corrected *p* value (FWE)	Cluster size (voxel)
1	Caudate (R:361)/Anterior cingulum (R:590)/Middle cingulum (R:10)/Inferior frontal operculum (R:177)/Inferior frontal orbital area (R:529)/Inferior frontal triangular (R:717)/Middle frontal medial area (R:219)/Middle frontal orbital area (R:62)/Middle frontal other areas (R:559)/Superior frontal medial area (R:1000)/Superior frontal orbital area (R:254)/Superior frontal other areas (R:2083)/Insula (R:232)/Pallidum (R:3)/Precentral gyrus (R:2)/Putamen (R:1003)/Rectus gyrus (L:3, R:594)/Rolandic operculum (R:170)/Supplemental motor area (R:109)/	Genu of corpus callosum (23)/Anterior limb of internal capsule (R:376)/Anterior corona radiata (R:290)/Superior corona radiata (R:141)/External capsule (R:632)/Cingulum (R:44)/Superior longitudinal fasciculus (R:235)/Superior fronto-occipital fasciculus (R:5)/Inferior fronto-occipital fasciculus (R:172)/	10.5	63	13.5	4.35	1569.00	0.010	11576
2	Amygdala (R:9)/Inferior temporal gyrus (R:81)/Middle temporal gyrus (R:345)/Temporal pole (R:355)/Superior temporal gyrus (R:42)/	Uncinate fasciculus (R:8)/	43.5	13.5	−27	4.13	1121.99	0.032	1009
3	Middle frontal orbital area (L:6)/Middle frontal other areas (L:331)/Superior frontal medial area (L:158)/Superior frontal orbital area (L:5)/Superior frontal other areas (L:343)/	None	−19.5	46.5	19.5	4.00	1088.65	0.035	877
4	Angular gyrus (R:20)/Middle cingulum (R:235)/Posterior cingulum (R:76)/Middle temporal gyrus (R:11)/	Body of corpus callosum (1)/Splenium of corpus callosum (29)/Posterior corona radiata (R:50)/Cingulum (R:145)/Superior longitudinal fasciculus (R:36)/	9	−28.5	37.5	3.78	1057.03	0.037	1113
5	Middle cingulum (R:4)/Paracentral lobule (R:17)/Superior parietal lobule	Superior corona radiata (R:1)/	19.5	−39	57	3.26	990.57	0.043	419
	(R:21)/Postcentral gyrus (R:79)/Precuneus (R:4)/								
6	Superior frontal medial area (L:17)/Superior frontal other areas (L:8)/	None	−10.5	66	15	3.32	960.36	0.048	25
7	Middle temporal gyrus (R:1)/	None	60	0	−27	2.91	944.48	0.050	1

*Most anatomical labels in gray matter were based on the WFU PickAtlas Tool (http://www.fmri.wfubmc.edu/cms/software#PickAtlas/) and on the PickAtlas automated anatomical labeling atlas option.

**The anatomical labels and significant clusters of major white matter fibers were determined using the ICBM DTI-81 Atlas (http://www.loni.ucla.edu/).

### Post-hoc analyses of associations between significant psychological correlates of hair mercury levels and mean values of anatomic clusters correlated with hair mercury levels

Partial correlation analyses controlling for the same covariates as in the main analyses were employed to examine associations between significant psychological correlates of hair mercury level and regional brain structure changes correlated with hair mercury level. Note that these types of post-hoc analyses are biased by the over-fitting effects in whole imaging analyses^29^ and so are presented for reference and as supplementary findings.

There were no significant results after corrections for multiple comparisons using FDR^30^. The statistical values are presented in Supplementary Table 2.

However, we previously reported significant correlations between total intelligence score on the TBIT and rGMV in whole-brain analyses and widespread positive correlations, including in an area of the left hippocampus, using a sample cohort overlapping with this study cohort^31^. Here, we found significant positive correlations between mean rGMV values of significant clusters and total intelligence score on the TBIT at the uncorrected level.

In addition, we previously reported significant correlations of rWMV with TBIT Perception score, Word-Color task score, and Color-Word task score, and found widespread white matter areas in all three analyses using a sample cohort overlapping with the current study cohort^32^. Consistently, here we found significant positive correlations between the mean rWMV signal of only two rWMV significant clusters and TBIT Perception score, Word-Color task score, and Color-Word task score at uncorrected level.

Therefore, in these regions and modalities, anatomical correlates of hair mercury level were partly overlapped with previously reported anatomical correlates of cognitive functions (and that are associated with hair mercury level in this study), and perhaps, observed psychological effects of hair mercury levels may be mediated by these brain areas and mechanisms.

## Discussion

In this study, we revealed associations of mercury levels as measured by hair analysis with neurocognitive functions and brain structures among a large sample of young adults in Japan, a country where fish consumption, the main source of mercury exposure, is relatively high. Consistent with our hypothesis, greater hair mercury levels were weakly but significantly associated with lower cognitive function scores, particularly on speed measures. However, in contrast to our hypothesis but consistent with several previous studies implicating low-level mercury exposure in depression, greater hair mercury was weakly associated with a lower depressive tendency. Also consistent with our hypothesis that greater hair mercury level is weakly but significantly associated with lower rGMV and rWMV, greater hair mercury level was weakly but significantly associated with lower rGMV in areas of the thalamus and hippocampus, as well as with a widespread lower rWMV. However, inconsistent with our hypothesis, greater hair mercury level was weakly associated with greater FA in bilateral white matter areas overlapping with areas of significant rWMV reductions, and weakly associated with lower MD in extensive gray and white matter areas, including widespread bilateral frontal lobe areas, right basal ganglia, right anterior temporal lobe, right posterior/middle cingulate cortex, and parietal cortex.

Utilizing a large sample of young healthy Japanese adults and hair mercury analysis, which is not affected by short-term fluctuations in intake, we show that greater chronic exposure is weakly but significantly associated with impaired cognitive functions (especially on tasks measuring processing speed). Previous studies have shown strong negative correlations between in utero mercury exposure and later cognitive function in the general population^6^ as well as more severe acute cognitive effects of high-level exposure (as in several public health disasters). However, studies on the effects of daily low-level exposure to mercury in adults using blood samples and urine samples have provided inconsistent results^7^. These differences could be due to the absence of effects on brain development and/or the full maturation of protective mechanisms such as the blood-brain barrier^13^. Further, compared to hair samples, blood and urine samples are strongly affected by temporal variations in mercury intake^33^. By resolving these problems through hair mercury analysis, we have demonstrated that even daily levels of mercury in the body are linked to low cognitive function in healthy young Japanese adults.

Using VBM, we also revealed weak but significant associations between greater hair mercury level and both lower rGMV in the thalamus/hippocampus and widespread reductions in rWMV. Similarly, body iron as measured by ferritin levels was associated with longitudinal rGMV reductions in areas of the hippocampus^34^. According to a previous review, one of the major mechanisms of methylmercury neurotoxicity is the generation of reactive oxygen species and ensuing oxidative stress^12^. The hippocampus is particularly vulnerable to oxidative stress^35^, which may explain the negative associations of hair iron levels and hippocampal GMV^34^. It is possible that the same mechanisms underline the present associations between hair mercury levels and rGMV. However, mercury is also known to inhibit the synthesis of DNA, RNA, and protein, and to promote the disassembly of cytoskeletal microtubules that maintain cellular three-dimensional structure and intracellular transport^12^. Further, it has also been suggested that accumulation of methylmercury in astrocytes inhibits glutamate uptake and leads to excitotoxicity^12^. These mechanisms of toxicity of mercury might lead to the decreased rGMV^12^.

In contrast to our original hypothesis, greater hair mercury level was weakly associated with greater FA in widespread white matter areas despite lower rWMV in overlapping areas. This finding is also inconsistent with studies showing that mercury has devastating effects on myelin and axon structure^11^. However, a rat study using diffusion-weighted imaging found that subacute methylmercury intoxication actually increased anisotropy, and electron microscopy revealed that this increased FA was associated not with demyelination (myelin was relatively undamaged in this study) but with loss of microtubules and neurofilaments in the axon, which normally restrict longitudinal water diffusion within the axoplasm and generate faster axonal flow, thus causing greater anisotropy^36^. The same mechanisms may partly explain the present findings. Alternatively, cytoskeletal disorganization caused by disassembly of microtubules, inhibition of DNA, RNA, and protein synthesis and associated loss of synapse and other structures, and glutamate excitotoxicity may reduced rWMV. Further, in a previous study, we reported that a wide range of simple processing speed measures were negatively correlated with rWMV in white matter areas distributed across the entire brain^32^. Consistently, in the present study, psychological correlates of hair mercury level (Total score of, Word-Color task, Color-Word task) are significantly and positively correlated with mean rWMV of the largest significant cluster after controlling age, sex and hair mercury level (all *P* < 0.02). Therefore, the present findings of negative associations between hair mercury levels and rWMV may explain the negative associations of hair mercury levels with performance on processing speed measures.

Contrary to predictions, greater hair mercury level was weakly associated with lower depression tendency. The reasons for this rather unexpected finding are not clear, especially considering that high-level exposure has been associated with depression^4^. However, as is the case for cognitive functions, the associations between blood or urine mercury levels and depression among the general population have varied among studies^9,10^. For instance, two such studies reporting no association^9,10^, while a larger-scale study of 6911 samples reporting a negative association (i.e., greater blood mercury levels correlated with lower odds of depression) in older adults (the same tendency is seen in young adults as well)^8^. In addition, animal studies have reported an association between greater methylmercury levels in the brain and inhibition of monoamine uptake^37^ or monoamine oxidase^38^, both of which are supposed to have anti-depressant effects^39^. Therefore, one speculation is that although high-level mercury exposure can cause neurodegeneration, leading to cognitive dysfunction and depression, lower-level daily exposure (albeit relatively high in Japan) has anti-depressant effects mediated by effects on monoamine systems. However, these are speculations and future studies are required to clarify these complex relationships between mercury exposure and depressive tendencies.

Another unexpected result was the negative correlation between MD and hair mercury level. As explained in the Introduction, various tissue components prevent free water diffusion, and thus lower MD; therefore, we hypothesized that greater hair mercury concentration would be associated with greater MD due to tissue destruction (i.e., reduced densities of tissue elements such as neurons). There are, however, a few plausible explanations for the observed negative association. One is lower cerebral blood flow in subjects with greater hair mercury. MD is known to be lowered by lower cerebral blood flow, too^40^. Previous studies have shown that high-level methylmercury exposure reduces cerebral blood flow prior to cerebral atrophy^41,42^. Another possibility is that methylmercury affects monoamine uptake. A previous study reported that facilitated dopaminergic function (as evidenced by relevant personality traits and greater dopamine synthesis capacity) was associated with lower MD, particularly in the dopaminergic system^19^. This could be due to an increase in the density of dopaminergic synapses or the iron loaded in the dopamine neurovesicles may lower MD^19^. This negative association of MD with hair mercury concentration, particularly in dopaminergic projection areas like the basal ganglia, may be relevant to the aforementioned association with monoamine dynamism and mercury. However, these explanations are highly speculative and require further study.

The associations revealed in this study require careful interpretation due to the generally small effect sizes. As discussed in our previous study^35^, whole-brain analyses tend to overestimate effect sizes, especially in small samples, due to over-fitting.^35^ Thus, individual small sample studies tend to have greater effect sizes than meta-analyses^37^. In the current study, several hundred individual datasets were included in each analysis but the observed effects sizes were relatively small (|partial correlation coefficients|< 0.18). As described above, however, true effect size, particularly for whole-brain analyses, can become smaller. A previous meta-analysis also reported small effect sizes (|*r*|< 0.16) for the association between body concentration of lead (a well-known neurotoxin) and individual cognitive differences^2^. Therefore, the present results are in accord with previous findings on the cognitive effects of trace heavy metals. However, the correlations between the concentration of trace heavy metals and neurocognitive outcomes may be weakened by the magnitude of measurement error in the concentration of trace heavy metals and those outcomes. It is also possible that the small variation in the concentration of heavy metals in hair among the normal population may have weakened those correlations. Thus, those weak correlations do not necessarily mean that trace heavy metals have only weak effects on the central nervous system.

The present study has a few limitations. First, this study focused on well educated young adults, so relevance to other populations must be confirmed in future studies. Further, this is a cross-sectional epidemiological study, so causal relationships could not be established. Other approaches such as longitudinal studies are necessary to establish causality. Finally, although we corrected for effects of family socioeconomic status and frequency of fish intake through multiple regression analyses, confounding effects may not be completely regressed out. For instance, certain intake parameters such as the total amount of fish eaten, species, and source may explain findings such as the associations between better mood state and hair mercury levels.

In conclusion, we utilized hair analysis and investigated how low-level mercury levels influence cognitive functions, rGMV, rWMV, FA, and MD in a large sample of young adults. The neurotoxic effects of high-level mercury exposure are severe, particularly on the developing brain and are well described due to several public health crises, such as those reported in Minamata, Japan, and in Iraq. Alternatively, studies on the effects of low-level mercury exposure as measured by blood or urine samples on cognitive functions among adults of the general population have yielded inconsistent results. In this study, using more stable hair mercury levels, routine accumulation was weakly associated with (a) lower rGMV in the left thalamus and left hippocampus, (b) lower rWMV in widespread areas, (c) greater FA of bilateral white matter tracts, (d) lower MD in widespread areas, particularly bilateral frontal lobe areas and the right basal ganglia, (e) poorer cognitive performance, particularly on tasks requiring cognitive speed, and (f) better mood state (less susceptibility to depressive states). These results suggest that even low-level exposure to mercury (mainly from fish consumption) is weakly and negatively associated with regional brain structures and neurobehavioral functions among young adults in Japan. Future studies using other approaches such as longitudinal analyses are warranted.

## Methods

### Subjects

The present study, which is a part of an ongoing project to investigate the associations among brain imaging characteristics, cognitive functions, and aging, included 920 healthy, right-handed individuals (561 males and 359 females) from whom hair Hg measurements and the data necessary for whole-brain imaging analyses were collected. The mean age of the subjects was 20.7 years [standard deviation, 1.8; age range: 18–27 years old]. All subjects were university students, postgraduates, or university graduates of less than one year’s standing. All subjects had normal vision and none had neurological or psychiatric illnesses according to self-reports. Handedness was evaluated using the Edinburgh Handedness Inventory^43^. Some of the subjects who took part in this study also became subjects of our interventional studies (psychological data and imaging data recorded before the intervention were used in this study)^44^. Psychological tests and magnetic resonance imaging (MRI) scans not described in this study were performed together with those described in this study. This study was approved by the Ethics Committee of Tohoku University. All procedures involving human participants were in accordance with the ethical standards of the institutional and/or national research committee and with the 1964 Helsinki declaration and its later amendments or comparable ethical standards.

Data analyses of each measure were conducted using the data of the sample from whom all the dependent and independent measures were properly obtained.

Subjects were instructed to get sufficient sleep, maintain their normal routines, eat sufficient breakfast, and to consume their normal amounts of caffeinated foods and drinks on the day of cognitive tests and MRI scans. In addition, subjects were instructed to avoid alcohol the night before the assessment. The descriptions in this subsection were mostly reproduced from another study of ours from the same project using the exactly same methods^45^.

### Details of recruitment and exclusion criteria of subjects

They were recruited using advertisements on bulletin boards at Tohoku University or via email introducing the study. These advertisements and emails specified the unacceptable conditions in individuals with regard to participation in the study such as handedness, the existence of metal in and around the body, claustrophobia, the use of certain drugs, a history of certain psychiatric and neurological diseases, and previous participation in related experiments.

A history of psychiatric and neurological diseases and/or recent drug use was assessed using our laboratory’s routine questionnaire, in which each subject answered questions related to their current or previous experiences of any of the listed diseases and listed drugs that they had recently taken. Drug screening was performed to confirm that the subjects were not taking any illegal psychostimulants or antipsychotic drugs, which was one of the exclusion criteria used during the course of the recruitment. Subjects with exclusion criteria should have been excluded before they came to the lab, but if they came for some reason, they had to go back once it was found that they met an exclusion criterion. Consequently, none had a history of neurological or psychiatric illness. In the course of this experiment, the scans were checked for obvious brain lesions and tumors, but there were no subjects having such obvious lesions or tumors.

These descriptions are mostly obtained from our previously published work^46^.

### Informed consent

Informed consent was obtained from all individual participants included in the study. Written informed consent was obtained from each subject. For nonadult subjects, written informed consent was obtained from parents or guardians.

### Hair acquisition and hair mineral analysis

As summarized in our previous studies^47^, hair mineral analysis is considered a reliable indicator of basic element levels in the body and has garnered considerable interest in multiple fields for summary, see^48^. Although the accuracy of hair analysis has been challenged, new preanalytic and analytic methods and good laboratory practices have improved precision^49^ and it has been suggested that consistent results can be obtained through sampling by trained personnel, standardized preanalytical and analytical procedures, and using suitable and sensitive equipment^14^. While blood and tissue testing also provide valuable information, hair is recognized as a potential repository of all elements that enter the body, and levels of elements in the hair indicate mineral accumulation over several months to years^50^. Serum levels of elements are maintained at the expense of tissue levels even in serious illnesses^33^. Excess minerals are quickly removed from the blood and deposited in tissues, including hair^33^. Thus, hair element levels are not subjected to rapid fluctuations of mineral intake and have long-term stability compared to blood and urine analyses^51^.

For mercury accumulation, hair is regarded as the preferred matrix for biomonitoring (for review, see^14^) and hair is a meaningful and representative tissue for biological monitoring of exposure to mercury^15^. Indeed, strong correlations have been found between hair values and environmental exposure (for review, see^14^).

Scalp hair samples (approximately 4 cm in length, 0.1 g in weight) were collected from each subject, with the hair cut as close to the scalp as possible. The hair samples were sent to a research laboratory (La Belle Vie Inc., Tokyo, Japan) and analyzed by established methods^52–57^ as described below.

Hair samples (75 mg) were weighed into 50-mL plastic tubes and washed twice, first with acetone and then with 0.01% Triton solution, in accordance with the procedures recommended by the Hair Analysis Standardization Board^58^. The washed hair sample was mixed with 10 ml of 6.25% tetramethylammonium hydroxide (Tama Chemical, Tokyo, Japan) and 50 μL of 0.1% gold solution (SPEX Certi Prep. Metuchen, NJ, USA), and then dissolved at 75 °C with shaking for 2 h. After cooling the solution to room temperature, an internal standard solution (containing Sc, Ga, and In) was added and the volume adjusted gravimetrically. Mineral concentrations were measured by inductively coupled plasma mass spectrometry (Agilent-7500ce; Agilent Technologies, Tokyo, Japan) using the internal standard method^52,59,60^ and are expressed in μg/g hair (ppm). For quality control, certified reference human hair samples supplied by the National Institute for Environmental Studies of Japan (Tsukuba, Japan; NIES CRM no. 13)^61^ were also analyzed. For statistical analysis, raw mercury concentrations were converted to logarithms as reported in the previous studies^47,52,55^.

We also recorded the last time the hair was colored, styled (‘permed’), or bleached for most subjects and divided answers as follows: (a) within 1 month, (b) within 1–2 months, (c) within 2–3 months, (d) within 3–6 months, and (e) not within 6 months. Based on hair growth rate and the sample length, the answers were coded as follows: (a) = 3, (b) = 2, (c) = 1, (d) = 0.5, and (e) = 0. We then investigated the associations of these values with mercury (Hg) levels in the hair after correcting for sex through partial correlation analyses. Although the history of hair perming showed a significant correlation, the effect size as a covariate was weak [partial correlation coefficient < 0.1, *P* = 0.039] compared to magnesium, calcium, selenium, and other factors [partial correlation coefficients of approximately 0.4], and therefore its effect was not considered. These additional analyses, including that of hair perm history as a covariate, did not substantially affect the strength of significant associations in the present study. The descriptions in this subsection were mostly reproduced from our previous study^45,47^, which used the similar methods.

The logarithms of mercury levels in the hair were used for all the analyses because logarithms of hair mercury levels were closer to the normal distribution and could alleviate the effects of outliers. This procedure of log-transformation has been performed in previous studies, including those from researchers affiliated with institutions in which mineral levels of our hair samples were measured (Research Laboratory, La Belle Vie Inc.)^52^.

### Neuropsychological tests and a questionnaire

The following neuropsychological tests and questionnaires were administered. We focused on processing speed measures due to previous studies documenting the effects of mercury on myelination (which is critical for the rapid transmission of neural signals) as well as on measures of affective mood states based on previous studies suggesting associations with depression (see Introduction). However, given the important contribution of processing speed on other cognitive functions, we also administered a wide range of neuropsychological tests to investigate their associations with hair mercury level in an exploratory manner.

These tests are described in this subsection and were largely reproduced from our previous studies e.g.,^62–65^.

[A] The Raven’s Advanced Progressive Matrices^66^ is a nonverbal reasoning task widely accepted as a reliable measure of general intelligence (for details, see our previous study;^67^. The test contains 36 nonverbal items requiring fluid reasoning ability. Each item consists of a 3 × 3 matrix with a missing piece to be completed by selecting the best of 8 alternatives. [B] Tanaka B-type intelligence test^68^. Type 3B, which is for examinees in their 3rd-year of junior high school and older, was used in this study. This test is a nonverbal mass intelligence test which does not include story problems but uses figures, single numbers, and letters as stimuli. In all subtests, subjects had to complete as many problems as possible within a certain time (a few minutes). This test consist of (a) a maze test (subjects had to trace a maze with a pencil from start to finish), (b) counting cubes (subjects had to count the number of cubes piled up in three-dimensional ways), (c) a displacement task (figures and numbers; subjects had to substitute a figure [9 figures] with a number [1 to 9] according to a model chart), (d) identification vs. same-different judgments (Japanese kana characters; subjects had to judge whether a pair of meaningless Japanese strings were the same), (e) filling in a sequence of numbers (subjects had to fill in the blanks of a number sequence with suitable numbers according to the rules of the number arrangement), (f) marking figures (subjects had to select forms which were identical to three samples from a series [sequence] of eight different forms), and (g) filling in figures (subjects had to complete uncompleted figures so that the uncompleted figures were the same as the sample figures when rotated). There are three TBIT subfactors, perception (tasks c, d, f), spatial relations (tasks a, b, g), and reasoning (task ee). The perception factor measures simple processing speed, the spatial relation factor measures spatial abilities to relate different things, and the reasoning factor measures reasoning abilities. [C] Two arithmetic tasks measured performance on two forms of one-digit times one-digit multiplication problem (a simple arithmetic task with numbers between 2 and 9) and two forms of two-digit times two-digit multiplication problem (a more complex arithmetic task with numbers between 11 and 19). The simple and complex arithmetic tasks had to be completed in 30 and 60 s, respectively. [D] The Stroop task (Hakoda’s version)^69,70^, which measures response inhibition and impulsivity. Hakoda’s version is a matching-type Stroop task requiring subjects to check whether their chosen answers are correct, unlike the traditional oral naming Stroop task. The test consists of two control tasks (Word-Color task, Color-Word task), a Stroop task and a reverse-Stroop task. In this study, we used the Word-Color and Color-Word tasks as measures of simple PS, and we used the Stroop and reverse-Stroop tasks as measures for inhibition. In the Word-Color task, a word naming a color (e.g. “red”) was presented in the leftmost of six columns. The other five columns were each filled with one of five colors and subjects had to check the column matching the written word in the leftmost column. In the Color-Word task, the leftmost of six columns was filled with a color and the other five columns contained written words naming different colors. Subjects had to check the column with the word matching the color of the leftmost column. In the reverse Stroop task, in the leftmost of six columns, a word naming a color was printed in another color (e.g., “red” was printed in blue letters) and the other five columns were each filled with five different colors from which subjects had to check the column whose color matched the written word in the leftmost column. In the Stroop task, in the leftmost of six columns, a word naming a color was printed in another color (e.g., “red” was printed in blue letters) and the other five columns contained words naming colors. Subjects had to check the column containing the word naming the color of the word in the leftmost column. In each task, subjects were instructed to complete as many of these exercises as possible in one minute. [E] A Japanese reading comprehension task developed by Kondo et al.^71^ was also included that required subjects to read articles and then answer four questions about the contents by choosing the correct response from five alternatives. For additional details on this test, such as how it was developed and its validity, refer to Kondo et al.^71^ and our previous study^62^. [F] The SA creativity test^72^ measures creativity through divergent thinking and involves three tasks (generate unique ways of using typical objects, imagine desirable functions for ordinary objects, and imagine the consequences of unimaginable things happening). [G] A (computerized) digit span task was used to assess working memory. Subjects were asked to view a progressively increasing number of random digits visually presented one-digit per second on a computer screen. They were then asked to repeat the sequence by pressing numbered buttons on the screen in the presented order (digit-span forward) or in the reverse order (digit-span backward), starting from two digits. Three sequences were given at each level until the participants responded incorrectly to all three sequences, at which point the task was ended. The score of each test is equal to the sum of the number of digits correctly repeated in the digit span forward and digit span backward tasks. [H] The Japanese version^73^ of the Beck Depression Inventory, was used as a measure of depression state.

### Image acquisition

The methods for magnetic resonance (MR) image acquisition were described in our previous study and reproduced below^45^. All MRI data were acquired using a 3-T Philips Achieva scanner. High-resolution T1-weighted structural images were collected using a magnetization-prepared rapid gradient-echo sequence (T1WIs: 240 × 240 matrix, TR = 6.5 ms, TE = 3 ms, FOV = 24 cm, slices = 162, slice thickness = 1.0 mm). Diffusion-weighted data were acquired using a spin-echo echo-planar imaging (EPI) sequence (TR = 10293 ms, TE = 55 ms, FOV = 22.4 cm, 2 × 2 × 2 mm^3^ voxels, 60 slices, SENSE reduction factor = 2, number of acquisitions = 1). The diffusion weighting was isotropically distributed along 32 directions (*b* value = 1,000 s/mm^2^). Additionally, three images with no diffusion weighting (*b* value = 0 s/mm^2^) (b = 0 images) were acquired using a spin-echo EPI sequence (TR = 10293 ms, TE = 55 ms, FOV = 22.4 cm, 2 × 2 × 2 mm^3^ voxels, 60 slices). FA and MD maps were calculated from the collected images using a commercially available diffusion tensor analysis package included in the MR console (Philips).

Diffusion images were acquired for phase correction and signal stabilization only and were not used as reconstructed images. Maps of MD and FA were calculated from the collected images using a commercially available diffusion tensor analysis package included with the MR console (Philips) as in many of our previous studies^21,74–77^. Furthermore, these image-generated results were congruent with those of previous studies^78,79^, confirming the validity of our analytic methods. These procedures involved correction for motion and distortion caused by eddy currents. Calculations were performed according to a standard method^80^. The quality of all imaging data was checked by visual inspection and images of low quality were excluded. Descriptions in this subsection were mostly reproduced from a previous study using similar methods^45^.

### Pre-processing of T1 weighted structural images

T1-weighted structural images were preprocessed using Statistical Parametric Mapping software (SPM12; Wellcome Department of Cognitive Neurology, London, UK) implemented in Matlab (Mathworks Inc., Natick, MA, USA). This was done for voxel-based morphometry analyses to measure rGMV and rWMV.

Preprocessing of structural data was performed using Statistical Parametric Mapping software (SPM12; Wellcome Department of Cognitive Neurology, London, UK) implemented in Matlab (Mathworks Inc., Natick, MA, USA). Using the new segmentation algorithm implemented in SPM12, T1-weighted structural images of each individual were segmented into 6 tissues. Default parameters were used in this new segmentation process, except that the Thorough Clean option was used to eliminate any odd voxel, affine regularization was performed with the International Consortium for Brain Mapping template for East Asian brains, and the sampling distance was set at 1 mm. We then proceeded to the diffeomorphic anatomical registration through exponentiated lie algebra (DARTEL) registration process implemented in SPM12. We used DARTEL import images of the 2 tissue probability maps from the aforementioned new segmentation process. First, the template for the DARTEL procedures was created using imaging data from 800 participants (400 males and 400 females). Next, the DARTEL procedures were performed for all subjects using this template and default parameter settings. The resulting images were spatially normalized to Montreal Neurological Institute space to yield images with 1.5 × 1.5 × 1.5 mm^3^ voxels. In addition, we performed a volume change correction (modulation) by modulating each voxel with the Jacobian determinants derived from spatial normalization, which allowed us to determine regional differences in the absolute amount of brain tissue^81^. Subsequently, all images were smoothed by convolving them with an isotropic Gaussian kernel of 8-mm full-width at half maximum (FWHM). The description in this paragraph was mainly reproduced from our previous study that used the same method^82^.

### Preprocessing procedures for FA and MD maps

Preprocessing and analysis of diffusion imaging data were performed using SPM8 implemented in Matlab. After correction for motion and distortion caused by eddy currents, artifactual images were removed by visual inspection as described in the Image Acquisition subsection. We normalized FA and MD images using a previously validated two-step segmentation process^21^. This process employs both MD and FA maps and a DARTEL-based registration process that also utilizes the FA signal distribution within the white matter area in the normalization procedure to yield images with 1.5 × 1.5 × 1.5 mm^3^ voxels. From the normalized FA images, voxels not likely to be white matter were then carefully removed and smoothed by convolution with an isotropic Gaussian kernel of 6-mm full-width at half maximum. From the normalized MD images, voxels not likely to be gray or white matter were then carefully removed, and the modified images smoothed by convolution with an isotropic Gaussian kernel of 8-mm full-width at half maximum. The description in this subsection was mostly reproduced from our previous study that used the same method^45^.

The details of these procedures are as follows. The descriptions are mostly reproduced from our previous study, which used the exact same methods^21^. Using the new segmentation algorithm implemented in SPM8, FA images of each individual were segmented into six tissues (first new segmentation). The default parameters and tissue probability maps were used in this process, except that affine regularization was performed using the International Consortium for Brain Mapping template for East Asian brains and the sampling distance (approximate distance between sampled points when estimating the model parameters) was 2 mm. We then synthesized the FA image and MD map. In the synthesized image, the area with a WM tissue probability >0.5 in the abovementioned new segmentation process was the FA image multiplied by −1 (hence, the synthesized image shows very clear contrast between WM and other tissues); the remaining area is the MD map (for details of this procedure, see below). The synthesized image from each individual was then segmented using the new segmentation algorithm implemented in SPM8 with the same parameters as above (second new segmentation). This two-step segmentation process was adopted because the FA image has a relatively clear contrast between GM and WM, as well as between WM and CSF, and the first new segmentation step can segment WM from other tissues. On the other hand, the MD map has clear contrast between GM and CSF and the second new segmentation can segment GM. Since the MD map alone lacks clear contrast between WM and GM, we must use a synthesized image (and the two-step segmentation process).

We then proceeded to the DARTEL registration process implemented in SPM8. We used the DARTEL import image of the GM tissue probability map produced in the second new segmentation process as the GM input for the DARTEL process. The WM input for the DARTEL process was created as follows. First, the raw FA image was multiplied by the WM tissue probability map from the second new segmentation process within the areas with a WM probability >0.5 (signals from other areas were set to 0). Next, the FA image * WM tissue probability map was coregistered and resliced to the DARTEL import WM tissue probability image from the second segmentation. The template for the DARTEL procedures was created using imaging data from 63 subjects who participated in the experiment in our lab^65^ and were included in the present study (meaning that they have the same characteristics as the subjects in this study). The first reason why we created the DARTEL template from the images of 63 subjects in the project and not from all subjects in the present study is because this is a large sample for creating a template compared to previous studies and thus cannot be considered problematic. The second reason is that the project in which the subjects participated is ongoing, and the DARTEL processes—especially our processes—require vast amounts of time and the resultant images require large storage resources; thus, we cannot reprocess the images of all subjects and add newer images whenever we change the number of subjects. Next, using this existing template, the DARTEL procedures were performed for all subjects in this study. In these procedures, the parameters were changed as follows to improve accuracy. The number of Gauss–Newton iterations performed within each outer iteration was set to 10 and, in each outer iteration, we used eightfold more timepoints to solve the partial differential equations than the default values. The number of cycles used by the full multi-grid matrix solver was set to 8. The number of relaxation iterations performed in each multi-grid cycle was also set to 8. The resultant synthesized images were spatially normalized to Montreal Neurological Institute space. Using these parameters, the raw FA map, MD map, rGMD, rWMD and rCSFD map from the abovementioned second new segmentation process were normalized to give images with 1.5 × 1.5 × 1.5 mm^3^ voxels. The FA image * WM tissue probability map was used in the DARTEL procedures because it includes different signal intensities within WM tissues and the normalization procedure can take advantage of intensity differences to adjust the image to the template from the perspective of the outer edge of the tissue and within the WM tissue. No modulation was performed in the normalization procedure.

The voxel size of the normalized FA images, MD images, and segmented images was 1.5 × 1.5 × 1.5 mm^3^.

Next, we created average images of normalized rGMD and rWMD images from the normalized rGMD and rWMD images from the subset of the entire sample (63 subjects)^21^. From the average image of normalized WM segmentation images from the 63 subjects mentioned above, we created mask image consisting of voxels with a WM signal intensity > 0.99. We then applied this mask image to the normalized FA image, thereby only retaining areas highly likely to be white matter. These images were smoothed (6 mm full-width half-maximum) and carried through to the second-level analyses of FA. As described previously^21^, through application of the mask, images unlikely to be WM or border areas between WM and other tissues were removed. The FA images were not affected by signals from tissues other than WM even after smoothing. This is important considering that, in these areas, WM volume and FA are highly correlated^20^ and the FA map supposedly reflects the extent of WM. Further, differences in rWMD compared with other tissues among individuals can be ignored after application of this mask because, within the masks, all voxels show very high white mater probability.

For analyses of MD images, we first created images from the normalized (a) MD, (b) rGMD, and (c) rCSFD maps in which areas not highly likely to be gray or white matter in our averaged normalized rGMD and rWMD images (defined by “gray matter tissue probability + white matter tissue probability < 0.99”) were removed (to exclude the strong effects of CSF on MD throughout analyses). These images were then smoothed (8-mm FWHM) and carried through to the second-level analyses of MD.

For validation of these preprocessing methods and comparison with other methods, see the supplementary online material of our previous study^21^. Briefly, in our previous study we demonstrated that this preprocessing procedure substantially lowers the deviation of normalized images from the template image and achieves better alignment to the template compared to ordinal normalization procedures. The congruence of findings obtained by tract-based spatial statistics and our preprocessing method in the basic analyses (effects of sex in the small sample) was shown in our previous study^21^.

Through these procedures, we believe that we successfully mitigated or removed the problems of voxel-based analysis of FA analysis raised by Smith et al^83^. These problems include (a) misalignment within white matter tissue (addressed by new segmentation processes and DARTEL processes that utilized difference in signal distribution within white matter using the FA signal) and (b) the effects of different tissue types and partial volume effects (addressed by new segmentation processes, the DARTEL processes, and application of the mask confined to images highly likely to be white matter (in the case of MD maps, white matter or gray matter)). Through these methods, the white matter of DTI images, as well as the gray matter areas of DTI images become available for analysis.

We avoided co-registration of DTI images to T1-weighted structural images because the shapes differ due to the unignorable distortion of EPI images in 3 T MRI.

### Statistics and reproducibility

#### Statistical analyses of psychological analyses

Psychological and non-whole brain imaging data were analyzed using Predictive Analysis Software, version 22.0.0 (SPSS Inc., Chicago, IL, USA; 2010). The associations of hair mercury level with psychological outcome variables were tested using partial correlation analyses. Control variables were sex, age, self-reported height, self-reported weight, and BMI (calculated from the self-reported height and self-reported weight), annual family income, parents’ highest educational qualifications (measured as reported previously;^28^ fatty fish intake, and intake of fish with less fat.

In these analyses, results with a threshold *P* < 0.05 (two-sided) were considered statistically significant after correcting for the false discovery rate using the graphically sharpened method^30^. This correction for multiple comparisons was performed among the 15 partial correlation analyses listed in Table 1. The sample sizes for the statistical analyses involving each psychological indicator are listed in Table 1.

#### Whole-brain statistical analysis

We investigated if the rGMV, rWMV, FA, and MD were associated with individual differences in the hair mercury level. The statistical analyses of imaging data were performed using SPM8. In these analyses of rGMV, rWMV, FA and MD, we performed whole-brain multiple regression analyses including sex, age, self-reported height, self-reported weight, BMI, which was calculated from the self-reported height and self-reported weight, family annual income, parents’ highest educational qualifications which were measured as has been reported previously^28^, amount of intake of fatty fish, intake of fish with less fat, and Hg levels in the hair. Fish intake was classified as follows (1, none; 2, less than once per week; 3, once per week; 4, two to three times per week; 5, 4 to 6 times per week; 6, once a day, 7, more than once a day) and used in the same way in the analysis. Note the education length of participants themselves is not necessary as all participants are students and they all start going to school at age of 6. The sample sizes for the statistical analyses involving VBM and DTI data are 920 and 919, respectively.

We included only voxels with a rGMV (or rWMV) signal intensity > 0.05 for all participants in the final analyses (meaning voxels with less than 5% probability of being gray (or white) matter in any participant were excluded from analyses). The FA analyses were limited to the mask of areas highly likely to be white matter (the mask of white matter tissue probability > 0.99 used in the preprocessing procedure). The MD analyses were limited to the mask of areas highly likely to be gray or white matter (the mask of gray matter tissue probability + white matter tissue probability > 0.99 used in the preprocessing procedure). The masks were created as described above.

A multiple comparison correction was performed using TFCE^84^ with randomized (5000 permutations) nonparametric testing using the TFCE toolbox (version:r64. http://dbm.neuro.uni-jena.de/tfce/). We applied a threshold of FWE corrected at *P* < 0.05 (this corresponds to one-sided test in SPM).

### Rationale for using SPM8 in the preprocessing of DTI data and statistical analyses

For preprocessing, we used SPM8 because our procedure is unique and has been validated only with this SPM version^21^. Furthermore, when we use SPM12 and the same parameter sets validated in SPM8, tissue types in certain brain areas are repeatedly misclassified during the segmentation process **(**Supplementary Fig. 2**)**.

We used SPM8 for statistical analyses due to the compatibility of SPM8 with the home-maid script used to set up statistical analyses. The results of permutation tests are not supposed to be affected by the version of SPM.

However, to confirm the robustness of results to version of the software, we also ran analyses using SPM12 for preprocessing of DTI data, second-level analyses, and newest version of TFCE toolbox (version:r177). Here, we use default parameter settings for TFCE toolbox. The results were presented in Supplementary Fig. 3 and mostly results were not affected by the using the newest version of the software.

#### Choosing covariates

In these regression analyses, we included multiple related covariates for each characteristic, including height, weight, and BMI for body size, annual family income and parents’ highest educational qualifications for socio-economic status, fatty fish intake and intake of fish with less fat for fish intake, because we could not determine a priori which would be a critical cofounder. However, even when only one of the related covariates was chosen (e.g., BMI, family income, fatty fish intake), there was little effect on the strength of the observed associations. Comparisons of statistical values between the main analyses and such supplemental analyses are presented in Supplementary Data 2. Results of imaging analyses were compared by using mean values of clusters shown to be significant in the main analyses.

Mercury and fish intake is supposed to be correlated and in this study, greater hair mercury level was significantly correlated with both greater fatty fish intake and intake of fish with less fat (both, *P* < 0.001 (two-sided), simple correlation, *r* = 0.278, and *r* = 0.200, respectively). Therefore, to exclude the possibility that hair mercury level analyses are confounded by large variations in individual fish intake, we regressed out the effects of fish intake. We also conducted additional analyses that excluded fatty fish intake and intake of fish with less fat as covariates. These results are presented in Supplementary Data 3. Overall, results were not substantially altered by removal of these covariates. However, the results of brain volume analyses became somewhat weak when these covariates were excluded. This may be due to the fact that fish intake tended to positively correlate with brain volume as well as with hair mercury level, and not adjusting for this effect may weaken the negative associations between brain volume measures and hair mercury levels.

There were no significant correlations of logarithmized hair mercury levels with logarithmized levels of other trace elements in hair (all partial correlation coefficients < |0.2| after controlling for sex). Logarithmized hair arsenic levels showed the strongest correlation level (partial correlation coefficient 0.172), but adding hair arsenic level as a covariate had little effect on other correlations with hair mercury. All other partial correlations with hair trace elements were below |0.12| after controlling for sex.

#### Comparison of results of FA analyses with tract-based spatial statistic (TBSS) based method

We believe that our thorough normalization procedure using two-step segmentation process, DARTEL and rigorous removal of irrelevant tissue is superior to the TBSS method.

On the other hand, since the TBSS method^83^ is a more widely used method, we also performed FA analysis using the TBSS method (the same analysis cannot be performed for MD because it includes gray matter). From diffusion images, FA images are calculated and then preprocessed and skeletonized in the standard way of FSL and TBSS (fsl.fmrib.ox.ac.uk/fsl/tbss/). In this TBSS analysis, the statistical model is the same as that of SPM analysis.

This analysis of TBSS with statistical analysis of permutation using TBSS and TFCE produced the same statistical trends as our SPM analysis, but the results were insignificant. However, the mean FA values of TBSS’s skelton in the masks of significant clusters that were identified in SPM analysis, significantly and positively correlated with hair mercury levels after adjusting for the nine covariates included in the main analysis (*N* = 919, *p* = 0.001 (two-sided), *t* = 3.392 in the case of cluster 1 in Table 3, *p* = 0.003 (two-sided), *t* = 3.002 in the case of cluster 2 in Table 3 and *p* = 0.043 (two-sided), *t* = 2.029, in the case of cluster 3 in Table 3).

We also confirm that generally TBSS’s FA value is more strongly affected by regional CSF density within the ROI. For example, within the ROI masks of corpus callosum genu, body and splenium [created from ICBM DTI-81 Atlas (http://www.loni.ucla.edu/)], total CSF density of normalized CSF images (normalized through our SPM method) was more strongly correlated with mean FA of TBSS’s skelton in the ROI than mean FA of FA maps that were preprocessed (but not smoothed) through our SPM method (*N* = 919, genu: TBSS, *r* = −0.25, SPM, *r* = 0.07, body: TBSS, *r* = −0.38, SPM, *r* = −0.10, splenium TBSS, *r* = −0.30, SPM, *r* = −0.02). This is despite the normalization process is common in CSF images and SPM’s FA images. Therefore, despite congruence of results of two analyses, we believe results obtained from our method is more valid.

### Reporting summary

Further information on research design is available in the Nature Research Reporting Summary linked to this article.

## Supplementary information

Supplementary Information

Supplementary Data 1

Supplementary Data 2

Supplementary Data 3

Reporting Summary

Description of Additional Supplementary Files

## Data Availability

All the experimental data obtained in the experiment of this study can be made available upon reasonable request from the corresponding author, pending approval from the ethics committee of Tohoku University, School of Medicine. Supplementary Data 1 includes all the independent and dependent variables that were used to generate the residual plots of Figs. 1–5.

## Code availability

We used following software and versions SPM8, SPM12 (https://www.fil.ion.ucl.ac.uk/spm/software/), Predictive Analysis Software, version 22.0.0 and TFCE toolbox (version:r64 and r177. http://dbm.neuro.uni-jena.de/tfce/). Supplemental analyses for TBSS were conducted using Freesurfer (linux-centos6_x86_64-7.1.1-20200723-8b40551) and FSL (version:6.0.4).
