# Supplementary material for: Mercury levels in hair are associated with reduced neurobehavioral performance and altered brain structures in young adults
Source: Commun Biol. 2022 Jun 2;5:529. doi: 10.1038/s42003-022-03464-z (PMC9163068; doi:10.1038/s42003-022-03464-z)
Supplement: Supplementary file 1 — Supplementary Information [file 42003_2022_3464_MOESM1_ESM.pdf]

Supplemental online material

Supplementary Table 1. Demographics of study participants

	Male (N = 561)		Female (N = 359)	
Measure	Mean	SD	Mean	SD
Age	20.84	1.92	20.54	1.65
RAPM[a]	28.76	3.90	28.02	3.87
Log-Mercury	3.25	0.22	3.20	0.23
Raw hair mercury level (µg/g)	2.01	1.15	1.85	1.19
Total intelligence score of TBIT[b]	114.31	12.14	109.27	11.34
Perception score of TBIT	49.49	7.26	48.76	6.79
Spatial relation factor of TBIT	43.92	5.07	41.45	4.74
Reasoning factor of TBIT	20.89	2.76	19.1	2.68
Simple arithmetic	31.96	5.3	30.04	4.93
Complex arithmetic	7.05	3.94	6.8	2.86
Word-Color task	71.33	8.09	69.98	7.21
Color-Word task	52.43	7.04	52.67	6.34
Reverse Stroop task	59.92	8.31	59.73	8.06
Stroop task	48.30	7.70	49.45	6.70

Reading comprehension	14.13	4.49	13.69	4.68
S-A creativity test	37.01	10.68	38.67	10.23
Digit span	36.88	7.22	34.5	6.58
Beck depression Inventory	7.9	6.22	8.5	6.63

[a]Raven’s advanced progressive matrices (a general intelligence task). [b]Tanaka B-type intelligence test.

Supplementary Table 2. Associations between significant psychological correlates of hair mercury levels and mean values of anatomic clusters significantly correlated with hair mercury levels (Numbers indicate the sample size of the analyses, partial correlation coefficients, uncorrected p values, and p values that are corrected for FDR).

	Total intelligence score of TBIT	Perception score of TBIT	Word-Color task	Color-Word task	Beck Depression Inventory
rGMV only cluster	839, 0.083, 0.017(0.14)	839, 0.034, 0.32(0.514)	916, 0.021, 0.519(0.629)	916, 0.03, 0.362(0.529)	913, 0.019, 0.568(0.647)
rWMV cluster 1	839, 0.109, 0.002(0.089)	839, 0.081, 0.019(0.14)	916, 0.088, 0.008(0.102)	916, 0.089, 0.007(0.102)	913, -0.014, 0.68(0.724)
rWMV cluster 2	839, 0.098, 0.005(0.102)	839, 0.079, 0.022(0.14)	916, 0.071, 0.032(0.168)	916, 0.075, 0.023(0.14)	913, -0.024, 0.463(0.6)
FA cluster 1	838, -0.033, 0.333(0.514)	838, -0.038, 0.269(0.514)	915, -0.022, 0.512(0.629)	915, 0.003, 0.919(0.854)	912, 0.006, 0.847(0.833)
FA cluster 2	838, -0.031, 0.368(0.529)	838, -0.037, 0.29(0.514)	915, -0.011, 0.737(0.75)	915, 0.019, 0.557(0.647)	912, -0.035, 0.284(0.514)
FA cluster 3	838, 0.013, 0.698(0.724)	838, 0.017, 0.624(0.697)	915, -0.003, 0.934(0.854)	915, 0.032, 0.329(0.514)	912, -0.049, 0.137(0.434)
MD cluster 1	838, 0.042, 0.223(0.509)	838, 0.035, 0.314(0.514)	915, 0.043, 0.193(0.467)	915, 0.015, 0.643(0.705)	912, 0.047, 0.16(0.434)
MD cluster 2	838, 0.037, 0.279(0.514)	838, 0.038, 0.271(0.514)	915, 0.028, 0.405(0.563)	915, 0.003, 0.936(0.854)	912, 0.058, 0.081(0.328)

MD cluster 3	838, 0.028, 0.42(0.57)	838, 0.036, 0.297(0.514)	915, 0.034, 0.307(0.514)	915, -0.002, 0.96(0.854)	912, 0.074, 0.025(0.14)
MD cluster 4	838, 0.05, 0.144(0.434)	838, 0.038, 0.27(0.514)	915, 0.047, 0.156(0.434)	915, 0.023, 0.479(0.607)	912, 0.06, 0.068(0.298)
MD cluster 5	838, 0.053, 0.122(0.434)	838, 0.045, 0.197(0.467)	915, 0.069, 0.036(0.169)	915, 0.03, 0.372(0.529)	912, 0.047, 0.154(0.434)
MD cluster 6	838, 0.004, 0.916(0.854)	838, 0.027, 0.433(0.573)	915, 0.047, 0.158(0.434)	915, -0.002, 0.947(0.854)	912, 0.086, 0.009(0.102)
MD cluster 7	838, -0.014, 0.693(0.724)	838, -0.001, 0.974(0.854)	915, 0.045, 0.173(0.448)	915, -0.02, 0.543(0.644)	912, 0.008, 0.814(0.814)

Supplementary Figure 1.

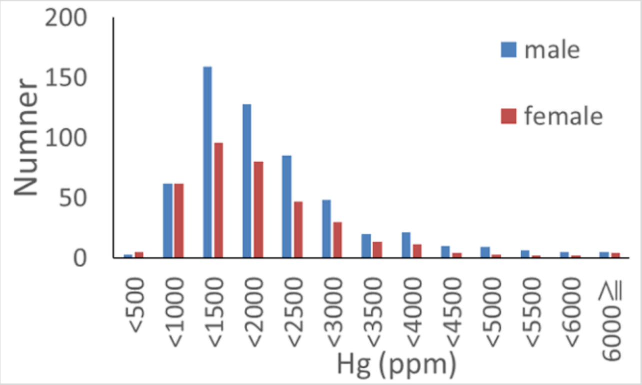

Supplementary Figure 1. The distribution of raw hair mercury levels in male and female subjects.

Supplementary Figure 2.

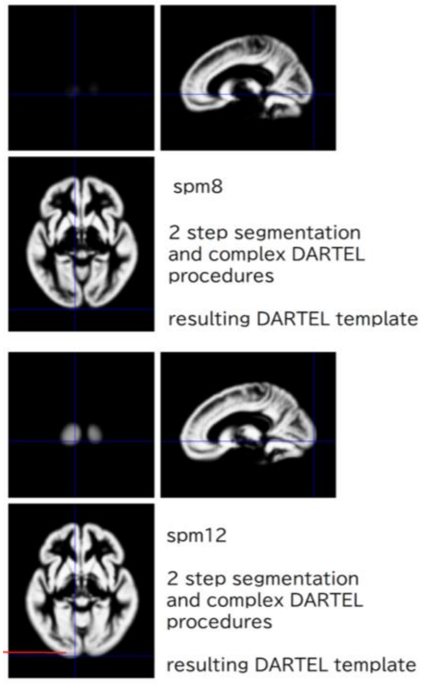

Supplementary Figure 2. The comparison of the DARTEL templates that are created from our preprocessing procedure using SPM 8 (upper) and SPM12 (lower). The figure of SPM 12 demonstrates the misclassification yielded by our modified preprocessing procedures. The red line denotes occipital regions which incorrectly recognized dura matter as gray matter when using SPM12.

Supplementary Figure 3.

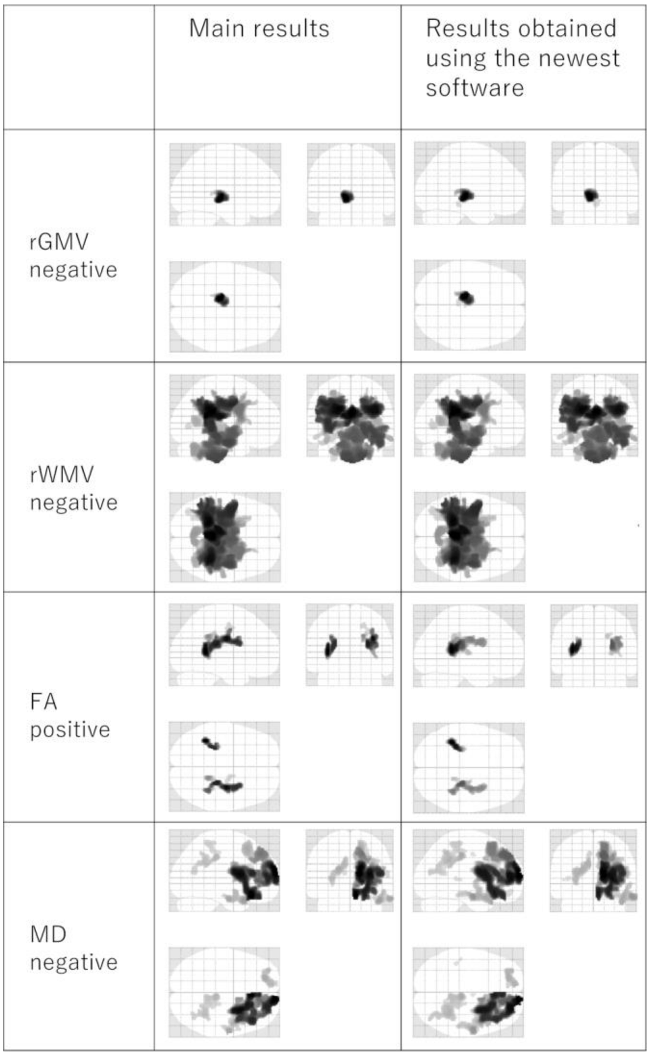

Supplementary Figure 3. Comparisons of significant results obtained through the main methods and significant results obtained using the newest version of software in all imaging procedures. The results shown were obtained using a threshold of TFCE, $P <$ 0.05 corrected (FWE) based on 5000 permutations. Regions of correlation are superimposed on a glass brain image of SPM. Significance of main results obtained through the method of the main analyses remained even when we use the newest

version of software in all imaging procedures.
