# Supplementary material for: Mercury levels in hair are associated with reduced neurobehavioral performance and altered brain structures in young adults
Source: Commun Biol. 2022 Jun 2;5:529. doi: 10.1038/s42003-022-03464-z (PMC9163068; doi:10.1038/s42003-022-03464-z)
Supplement: Supplementary file 5 — Reporting Summary [file 42003_2022_3464_MOESM5_ESM.pdf]

nature research

Corresponding author(s): Hikaru Takeuchi

Last updated by author(s): Apr 4, 2022

Reporting Summary

Nature Research wishes to improve the reproducibility of the work that we publish. This form provides structure for consistency and transparency in reporting. For further information on Nature Research policies, see our Editorial Policies and the Editorial Policy Checklist.

Statistics

For all statistical analyses, confirm that the following items are present in the figure legend, table legend, main text, or Methods section.

n/a	Confirmed	
☐	☒	The exact sample size (*n*) for each experimental group/condition, given as a discrete number and unit of measurement
☒	☐	A statement on whether measurements were taken from distinct samples or whether the same sample was measured repeatedly
☐	☒	The statistical test(s) used AND whether they are one- or two-sided<br>*Only common tests should be described solely by name; describe more complex techniques in the Methods section.*
☐	☒	A description of all covariates tested
☐	☒	A description of any assumptions or corrections, such as tests of normality and adjustment for multiple comparisons
☐	☒	A full description of the statistical parameters including central tendency (e.g. means) or other basic estimates (e.g. regression coefficient) AND variation (e.g. standard deviation) or associated estimates of uncertainty (e.g. confidence intervals)
☐	☒	For null hypothesis testing, the test statistic (e.g. *F*, *t*, *r*) with confidence intervals, effect sizes, degrees of freedom and *P* value noted<br>*Give P values as exact values whenever suitable.*
☒	☐	For Bayesian analysis, information on the choice of priors and Markov chain Monte Carlo settings
☒	☐	For hierarchical and complex designs, identification of the appropriate level for tests and full reporting of outcomes
☐	☒	Estimates of effect sizes (e.g. Cohen's *d*, Pearson's *r*), indicating how they were calculated

Our web collection on statistics for biologists contains articles on many of the points above.

Software and code

Policy information about availability of computer code

Data collection	No software for data collection.
Data analysis	Psychological data were analyzed using Predictive Analysis Software, version 22.0.0 (SPSS Inc., Chicago, IL, USA; 2010).<br>SPM8 was used for preprocessing and statistical analyses of whole brain neuroimaging data.<br>For the cross-sectional whole brain analyses, multiple comparison corrections were performed using threshold-free cluster enhancement (TFCE) 52 with randomized (5,000 permutations) nonparametric testing using the TFCE toolbox (version:r64, http://dbm.neuro.uni-jena.de/tfce/).

For manuscripts utilizing custom algorithms or software that are central to the research but not yet described in published literature, software must be made available to editors and reviewers. We strongly encourage code deposition in a community repository (e.g. GitHub). See the Nature Research guidelines for submitting code & software for further information.

Data

Policy information about availability of data

All manuscripts must include a data availability statement. This statement should provide the following information, where applicable:

- Accession codes, unique identifiers, or web links for publicly available datasets
- A list of figures that have associated raw data
- A description of any restrictions on data availability

All the experimental data obtained in the experiment of this study will be available to ones that were admitted in the ethics committee of Tohoku University, school of medicine. All the data sharing should be first admitted by the ethics committee of Tohoku University, school of medicine.

Field-specific reporting

Please select the one below that is the best fit for your research. If you are not sure, read the appropriate sections before making your selection.

☒ Life sciences ☐ Behavioural & social sciences ☐ Ecological, evolutionary & environmental sciences

For a reference copy of the document with all sections, see nature.com/documents/nr-reporting-summary-flat.pdf

Life sciences study design

All studies must disclose on these points even when the disclosure is negative.

Sample size	The present study, which is a part of an ongoing project to investigate the associations among brain imaging characteristics, cognitive functions, and aging, included 920 healthy, right-handed individuals (561 males and 359 females) from whom the data necessary for whole-brain analyses and hair Hg levels were collected.<br>The mean age of the subjects was 20.7 years [standard deviation (SD), 1.8; age range: 18–27 years old].
Data exclusions	Data analyses of each measure was conducted using the data of the sample from whom all the dependent and independent measures were properly obtained.
Replication	There are no replication analyses.
Randomization	Randomization procedures are irrelevant in this study.
Blinding	Blinding procedures are not irrelevant in this study.

Reporting for specific materials, systems and methods

We require information from authors about some types of materials, experimental systems and methods used in many studies. Here, indicate whether each material, system or method listed is relevant to your study. If you are not sure if a list item applies to your research, read the appropriate section before selecting a response.

Materials & experimental systems

n/a	Involved in the study	
☒	☐	Antibodies
☒	☐	Eukaryotic cell lines
☒	☐	Palaeontology and archaeology
☒	☐	Animals and other organisms
☐	☒	Human research participants
☒	☐	Clinical data
☒	☐	Dual use research of concern

Methods

n/a	Involved in the study	
☒	☐	ChIP-seq
☒	☐	Flow cytometry
☐	☒	MRI-based neuroimaging

Human research participants

Policy information about studies involving human research participants

Population characteristics	The present study, which is a part of an ongoing project to investigate the associations among brain imaging characteristics, cognitive functions, and aging, included 920 healthy, right-handed individuals (561 males and 359 females) from whom the data necessary for whole-brain analyses and hair Hg levels were collected. The mean age of the subjects was 20.7 years [standard deviation (SD), 1.8; age range: 18–27 years old].<br><br>All subjects were university students, postgraduates, or university graduates of less than one year's standing. All subjects had normal vision and none had neurological or psychiatric illnesses according to self-reports. Handedness was evaluated using the Edinburgh Handedness Inventory 28.
Recruitment	Details of recruitment and exclusion criteria of subjects<br>They were recruited using advertisements on bulletin boards at Tohoku University or via email introducing the study. These advertisements and emails specified the unacceptable conditions in individuals with regard to participation in the study such as handedness, the existence of metal in and around the body, claustrophobia, the use of certain drugs, a history of certain psychiatric and neurological diseases, and previous participation in related experiments.<br>A history of psychiatric and neurological diseases and/or recent drug use was assessed using our laboratory's routine questionnaire, in which each subject answered questions related to their current or previous experiences of any of the listed diseases and listed drugs that they had recently taken. Drug screening was performed to confirm that the subjects were not taking any illegal psychostimulants or antipsychotic drugs, which was one of the exclusion criteria used during the course of the recruitment. Subjects with exclusion criteria should have been excluded before they came to the lab, but if they came for

some reason, they had to go back once it was found that they met an exclusion criterion. Consequently, none had a history of neurological or psychiatric illness. In the course of this experiment, the scans were checked for obvious brain lesions and tumors, but there were no subjects having such obvious lesions or tumors.
These descriptions are mostly obtained from our previously published work 4.

Ethics oversight

Approval for these experiments was obtained from the Institutional Review Board of Tohoku University.

Note that full information on the approval of the study protocol must also be provided in the manuscript.

Magnetic resonance imaging

Experimental design

Design type

Cross-sectional observation study

Design specifications

We investigated the associations of hair lead levels with cognitive measures, regional gray matter and white matter volume, and FA of the white matter in a large cohort of typically developing young adults.

Behavioral performance measures

Psychological measures
The following neuropsychological tests and questionnaires were administered. We focused on processing speed measures due to previous studies documenting effects of mercury on myelination (which is critical for the rapid transmission of neural signals) as well as on measures of affective mood states based on previous studies suggesting associations with depression (see Introduction). However, given the important contribution of processing speed on other cognitive functions, we also administered a wide range of cognitive functions to investigate their associations with hair mercury level in an exploratory manner.
These tests are described in this subsection and were largely reproduced from our previous studies e.g., 36. [A] The Raven's Progressive Matrices (RAPM, 37 is a non-verbal reasoning task widely accepted as a reliable measure of general intelligence (for details, see our previous study; 38. [B] The Tanaka B-type intelligence test 39 type 3B (TBIT) is a non-verbal mass intelligence test used for 3rd-year junior high school and older examinees in Japan. It does not include story problems but rather uses figures, single numbers, and letters as stimuli. In all subtests, the subjects have to solve as many problems as possible within a certain time (a few minutes), meaning these are complex cognitive speed tasks. For more details, see our previous study 40. There are three TBIT subfactors, perception, spatial relations, and reasoning. The perception factor measures simple processing speed, the spatial relation factor measures spatial abilities to relate different things, and the reasoning factor measures reasoning abilities. [C] Two arithmetic tasks measured performance on two forms of one-digit times one-digit multiplication problem (a simple arithmetic task with numbers between 2 and 9) and two forms of two-digit times two-digit multiplication problem (a more complex arithmetic task with numbers between 11 and 19). The simple and complex arithmetic tasks had to be completed in 30 and 60 s, respectively. [D] The Stroop task (Hakoda's version) 41 measures response inhibition and impulsivity. Hakoda's version is a matching-type Stroop task requiring subjects to check whether their chosen answers are correct, unlike the traditional oral naming Stroop task. The test consists of two control tasks (Word-Color and Color-Word tasks), a Stroop task, and a reverse Stroop task (see our previous study for details; 42. [E] The reading comprehension task used was developed by Kondo et al. 43. For more details on this test, such as how it was developed and its validity, refer to Kondo et al. 43 and our previous study 36. [F] The S-A creativity test measures creativity by divergent thinking 44. [G] A (computerized) digit span task was used as a working memory task for details, see 45. [H] The Japanese version 46 of the Beck Depression Inventory, was used as a measure of depression state.

Acquisition

Imaging type(s)

Diffusion-weighted data and T1-weighted structural image

Field strength

3T

Sequence & imaging parameters

High-resolution T1-weighted structural images were collected using a magnetization-prepared rapid gradient echo sequence (T1WIs: 240 × 240 matrix, TR = 6.5 ms, TE = 3 ms, FOV = 24 cm, slices = 162, slice thickness = 1.0 mm). Diffusion-weighted data were acquired using a spin-echo EPI sequence (TR = 10293ms, TE = 55 ms, FOV = 22.4 cm, 2×2×2 mm3 voxels, 60 slices, SENSE reduction factor = 2, number of acquisitions = 1). The diffusion weighting was isotropically distributed along 32 directions (b value = 1,000 s/mm2). Additionally, three images with no diffusion weighting (b value = 0 s/mm2) (b = 0 images) were acquired using a spin-echo EPI sequence (TR = 10293 ms, TE = 55 ms, FOV = 22.4 cm, 2 ⍰ 2 ⍰ 2 mm3 voxels, 60 slices).

Area of acquisition

Whole-brain

Diffusion MRI ☒ Used ☐ Not used

Parameters

Described in sequence & imaging parameters

Preprocessing

Preprocessing software

SPM8 & 12

Normalization

Pre-processing of T1 weighted structural images. T1-weighted structural images were preprocessed using Statistical

Normalization

Parametric Mapping software (SPM12; Wellcome Department of Cognitive Neurology, London, UK) implemented in Matlab (Mathworks Inc., Natick, MA, USA). This was done for voxel-based morphometry analyses to measure rGMV and rWMV. Preprocessing of structural data was performed using Statistical Parametric Mapping software (SPM12; Wellcome Department of Cognitive Neurology, London, UK) implemented in Matlab (Mathworks Inc., Natick, MA, USA). Using the new segmentation algorithm implemented in SPM12, T1-weighted structural images of each individual were segmented into 6 tissues. Default parameters were used in this new segmentation process, except that the Thorough Clean option was used to eliminate any odd voxel, affine regularization was performed with the International Consortium for Brain Mapping template for East Asian brains, and the sampling distance was set at 1 mm. We then proceeded to the diffeomorphic anatomical registration through exponentiated lie algebra (DARTEL) registration process implemented in SPM12. We used DARTEL import images of the 2 tissue probability maps from the aforementioned new segmentation process. First, the template for the DARTEL procedures was created using imaging data from 800 participants (400 males and 400 females). Next, the DARTEL procedures were performed for all subjects using this template and default parameter settings. The resulting images were spatially normalized to Montreal Neurological Institute (MNI) space to yield images with 1.5�� 1.5 � 1.5 mm3 voxels. In addition, we performed a volume change correction (modulation) by modulating each voxel with the Jacobian determinants derived from spatial normalization, which allowed us to determine regional differences in the absolute amount of brain tissue (81). Subsequently, all images were smoothed by convolving them with an isotropic Gaussian kernel of 8-mm full-width at half maximum (FWHM). The description in this paragraph was mainly reproduced from our previous study that used the same method (82).

Preprocessing procedures for FA and MD maps.
Preprocessing and analysis of diffusion imaging data were performed using SPM8 implemented in Matlab. After correction for motion and distortion caused by eddy currents, artifactual images were removed by visual inspection as described in the Image Acquisition subsection. We normalized FA and MD images using a previously validated two-step segmentation process (21). This process employs both MD and FA maps and a DARTEL-based registration process that also utilizes the FA signal distribution within the white matter area in the normalization procedure to yield images with 1.5 � 1.5 � 1.5 mm3 voxels. From the normalized FA images, voxels not likely to be white matter were then carefully removed and smoothed by convolution with an isotropic Gaussian kernel of 6-mm full-width at half maximum. From the normalized MD images, voxels not likely to be gray or white matter were then carefully removed, and the modified images smoothed by convolution with an isotropic Gaussian kernel of 8-mm full-width at half maximum. The description in this subsection was mostly reproduced from our previous study that used the same method (45).
The details of these procedures are as follows. The descriptions are mostly reproduced from our previous study, which used the exact same methods (21). Using the new segmentation algorithm implemented in SPM8, FA images of each individual were segmented into six tissues (first new segmentation). The default parameters and tissue probability maps were used in this process, except that affine regularization was performed using the International Consortium for Brain Mapping template for East Asian brains and the sampling distance (approximate distance between sampled points when estimating the model parameters) was 2 mm. We then synthesized the FA image and MD map. In the synthesized image, the area with a WM tissue probability >0.5 in the abovementioned new segmentation process was the FA image multiplied by −1 (hence, the synthesized image shows very clear contrast between WM and other tissues); the remaining area is the MD map (for details of this procedure, see below). The synthesized image from each individual was then segmented using the new segmentation algorithm implemented in SPM8 with the same parameters as above (second new segmentation). This two-step segmentation process was adopted because the FA image has a relatively clear contrast between GM and WM, as well as between WM and CSF, and the first new segmentation step can segment WM from other tissues. On the other hand, the MD map has clear contrast between GM and CSF and the second new segmentation can segment GM. Since the MD map alone lacks clear contrast between WM and GM, we must use a synthesized image (and the two-step segmentation process).
We then proceeded to the DARTEL registration process implemented in SPM8. We used the DARTEL import image of the GM tissue probability map produced in the second new segmentation process as the GM input for the DARTEL process. The WM input for the DARTEL process was created as follows. First, the raw FA image was multiplied by the WM tissue probability map from the second new segmentation process within the areas with a WM probability >0.5 (signals from other areas were set to 0). Next, the FA image * WM tissue probability map was coregistered and resliced to the DARTEL import WM tissue probability image from the second segmentation. The template for the DARTEL procedures was created using imaging data from 63 subjects who participated in the experiment in our lab (65) and were included in the present study (meaning that they have the same characteristics as the subjects in this study). The first reason why we created the DARTEL template from the images of 63 subjects in the project and not from all subjects in the present study is because this is a large sample for creating a template compared to previous studies and thus cannot be considered problematic. The second reason is that the project in which the subjects participated is ongoing, and the DARTEL processes—especially our processes—require vast amounts of time and the resultant images require large storage resources; thus, we cannot reprocess the images of all subjects and add newer images whenever we change the number of subjects. Next, using this existing template, the DARTEL procedures were performed for all subjects in this study. In these procedures, the parameters were changed as follows to improve accuracy. The number of Gauss–Newton iterations performed within each outer iteration was set to 10 and, in each outer iteration, we used 8-fold more timepoints to solve the partial differential equations than the default values. The number of cycles used by the full multi-grid matrix solver was set to 8. The number of relaxation iterations performed in each multi-grid cycle was also set to 8. The resultant synthesized images were spatially normalized to MNI space. Using these parameters, the raw FA map, MD map, rGMD, rWMD and rCSFD map from the abovementioned second new segmentation process were normalized to give images with 1.5�� 1.5 � 1.5 mm3 voxels. The FA image * WM tissue probability map was used in the DARTEL procedures because it includes different signal intensities within WM tissues and the normalization procedure can take advantage of intensity differences to adjust the image to the template from the perspective of the outer edge of the tissue and within the WM tissue. No modulation was performed in the normalization procedure.
The voxel size of the normalized FA images, MD images, and segmented images was 1.5 � 1.5 � 1.5 mm3.
Next, we created average images of normalized rGMD and rWMD images from the normalized rGMD and rWMD images from the subset of the entire sample (63 subjects) (21). From the average image of normalized WM segmentation images from the 63 subjects mentioned above, we created mask image consisting of voxels with a WM signal intensity > 0.99. We then applied this mask image to the normalized FA image, thereby only retaining areas highly likely to be white matter. These images were smoothed (6 mm full-width half-maximum) and carried through to the second-level analyses of FA. As described previously (21), through application of the mask, images unlikely to be WM or border areas between WM and other tissues were removed. The FA images were not affected by signals from tissues other than WM even after smoothing. This is important considering that, in these areas, WM volume and FA are highly correlated (20) and the FA map supposedly reflects

the extent of WM. Further, differences in rWMD compared with other tissues among individuals can be ignored after application of this mask because, within the masks, all voxels show very high white mater probability.
For analyses of MD images, we first created images from the normalized (a) MD, (b) rGMD, and (c) rCSFD maps in which areas not highly likely to be gray or white matter in our averaged normalized rGMD and rWMD images (defined by “gray matter tissue probability + white matter tissue probability < 0.99”) were removed (to exclude the strong effects of CSF on MD throughout analyses). These images were then smoothed (8-mm FWHM) and carried through to the second-level analyses of MD.
For validation of these preprocessing methods and comparison with other methods, see the supplementary online material of our previous study (21). Briefly, in our previous study we demonstrated that this preprocessing procedure substantially lowers the deviation of normalized images from the template image and achieves better alignment to the template compared to ordinal normalization procedures. The congruence of findings obtained by tract-based spatial statistics and our preprocessing method in the basic analyses (effects of sex in the small sample) was shown in our previous study (21).
Through these procedures, we believe that we successfully mitigated or removed the problems of voxel-based analysis of FA analysis raised by Smith et al (83). These problems include (a) misalignment within white matter tissue (addressed by new segmentation processes and DARTEL processes that utilized difference in signal distribution within white matter using the FA signal) and (b) the effects of different tissue types and partial volume effects (addressed by new segmentation processes, the DARTEL processes, and application of the mask confined to images highly likely to be white matter (in the case of MD maps, white matter or gray matter)). Through these methods, the white matter of DTI images as well as the gray matter areas of DTI images become available for analysis.
We avoided co-registration of DTI images to T1 weighted structural images because the shapes differ due to the unignorable distortion of EPI images in 3T MRI.

Normalization template

Described in "Normalization space". The standardized space is MNI305.

Noise and artifact removal

Diffusion images were acquired for phase correction and signal stabilization only and were not used as reconstructed images. Maps of MD and FA were calculated from the collected images using a commercially available diffusion tensor analysis package included with the MR console (Philips) as in many of our previous studies (15-19). Furthermore, these image-generated results were congruent with those of previous studies (20, 21), confirming the validity of our analytic methods. These procedures involved correction for motion and distortion caused by eddy currents. Calculations were performed according to a previously proposed method (22). The quality of all imaging data was checked by visual inspection and images of low quality were excluded.

Volume censoring

Visual inspection

Statistical modeling & inference

Model type and settings

Statistical analyses of psychological analyses
Psychological and non-whole brain imaging data were analyzed using Predictive Analysis Software, version 22.0.0 (SPSS Inc., Chicago, IL, USA; 2010). The associations of hair mercury level with psychological outcome variables were tested using partial correlation analyses. Control variables were sex, age, self-reported height, self-reported weight, and body mass index (BMI, calculated from the self-reported height and self-reported weight), annual family income, parents’ highest educational qualifications (measured as reported previously; (28) fatty fish intake, and intake of fish with less fat.
In these analyses, results with a threshold $P < 0.05$ (two-sided) were considered statistically significant after correcting for the false discovery rate using the graphically sharpened method (30). This correction for multiple comparisons was performed among the 15 partial correlation analyses listed in Table 1.

Whole-brain statistical analysis
We investigated if the rGMV, rWMV, FA and MD were associated with individual differences in hair mercury level. The statistical analyses of imaging data were performed using SPM8. In these analyses of rGMV, rWMV, FA and MD, we performed whole-brain multiple regression analyses including sex, age, self-reported height, self-reported weight, body mass index (BMI), which was calculated from the self-reported height and self-reported weight, family annual income, parents’ highest educational qualifications which were measured as has been reported previously 28, amount of intake of fatty fish, intake of fish with less fat, and Hg levels in the hair. Fish intake was classified as follows (1, none; 2, less than once per week; 3, once per week; 4, two to three times per week; 5, 4 to 6 times per week; 6, once a day, 7, more than once a day) and used in the same way in the analysis. Note the education length of participants themselves is not necessary as all participants are students and they all start going to school at age of 6.
We included only voxels with a rGMV (or rWMV) signal intensity > 0.05 for all participants in the final analyses (meaning voxels with less than 5% probability of being gray (or white) matter in any participant were excluded from analyses). The FA analyses were limited to the mask of areas highly likely to be white matter (the mask of white matter tissue probability > 0.99 used in the preprocessing procedure). The mask was created as described in Supplemental Methods.
A multiple comparison correction was performed using threshold-free cluster enhancement (TFCE) 84 with randomized (5,000 permutations) nonparametric testing using the TFCE toolbox (version:r64. http://dbm.neuro.uni-jena.de/tfce/). We applied a threshold of FWE corrected at $P < 0.05$ (this corresponds to one-sided test in SPM).

Rationale for using SPM8 in the preprocessing of DTI data and statistical analyses
For preprocessing, we used SPM8 because our procedure is unique and has been validated only with this SPM version 21. Furthermore, when we use SPM12 and the same parameter sets validated in SPM8, tissue types in certain brain areas are repeatedly misclassified during the segmentation process (Supplemental Fig. 2).
We used SPM8 for statistical analyses due to the compatibility of SPM8 with the home-maid script used to set up statistical analyses. The results of permutation tests are not supposed to be affected by the version of SPM.
However, to confirm the robustness of results to version of the software, we also ran analyses using SPM12 for preprocessing of DTI data, second-level analyses, and newest version of TFCE toolbox (version:r177). Here, we use default parameter settings for TFCE toolbox. The results were presented in supplemental Fig. 3 and mostly results were not affected by the using the newest version of the software.

Effect(s) tested	individual differences in hair mercury level (logarithm)

Specify type of analysis: ☒ Whole brain ☐ ROI-based ☐ Both

Statistic type for inference (See Eklund et al. 2016)	Described in model type and settings
Correction	Statistical analyses of non-whole-brain analyses<br>In these analyses, results with a threshold P < 0.05 (two-sided) were considered statistically significant after correcting for the false discovery rate using the graphically sharpened method 48. This correction for multiple comparisons was performed among the 15 partial correlation analyses listed in Table 1.<br><br>Whole-brain statistical analysis<br>A multiple comparison correction was performed using threshold-free cluster enhancement (TFCE) 50 with randomized (5,000 permutations) nonparametric testing using the TFCE toolbox (version:r64, http://dbm.neuro.uni-jena.de/tfce/). We applied a threshold of FWE corrected at P < 0.05 (this corresponds to one-sided test in SPM).

Models & analysis

n/a	Involved in the study
☒	☐ Functional and/or effective connectivity
☒	☐ Graph analysis
☒	☐ Multivariate modeling or predictive analysis
