# Supplementary material for: Mercury levels in hair are associated with reduced neurobehavioral performance and altered brain structures in young adults
Source: Commun Biol. 2022 Jun 2;5:529. doi: 10.1038/s42003-022-03464-z (PMC9163068; doi:10.1038/s42003-022-03464-z)
Supplement: Supplementary file 6 — Description of Additional Supplementary Files [file 42003_2022_3464_MOESM6_ESM.pdf]

Description of Additional Supplementary Files

File name: Supplementary Data 1
Description: The source data behind the graphs in the paper.

File name: Supplementary Data 2
Description: The table of comparisons of partial correlations between psychological variables and hair mercury level after correcting for different sets of confounding variables.

File name: Supplementary Data 3
Description: The table of comparisons of partial correlations between psychological variables and hair mercury level including (as in the main analyses) or excluding fish intake covariates.
